# Single nucleotide polymorphisms associated with wine fermentation and adaptation to nitrogen limitation in wild and domesticated yeast strains

**DOI:** 10.1186/s40659-023-00453-2

**Published:** 2023-07-29

**Authors:** Eduardo I. Kessi-Pérez, Eric Acuña, Camila Bastías, Leyanis Fundora, Manuel Villalobos-Cid, Andrés Romero, Sakshi Khaiwal, Matteo De Chiara, Gianni Liti, Francisco Salinas, Claudio Martínez

**Affiliations:** 1 Centro de Estudios en Ciencia y Tecnología de Alimentos (CECTA), Universidad de Santiago de Chile (USACH), Santiago, Chile; 2 Departamento de Ciencia y Tecnología de los Alimentos, Universidad de Santiago de Chile (USACH), Santiago, Chile; 3 Departamento de Ingeniería Informática, Program for the Development of Sustainable Production Systems (PDSPS), Facultad de Ingeniería, Universidad de Santiago de Chile (USACH), Santiago, Chile; 4 Laboratorio de Genómica Funcional, Instituto de Bioquímica y Microbiología, Facultad de Ciencias, Universidad Austral de Chile, Valdivia, Chile; 5 ANID-Millennium Science Initiative-Millennium Institute for Integrative Biology (iBio), Santiago, Chile; 6 Université Côte d’Azur, CNRS, Inserm, IRCAN, Nice, France

**Keywords:** Adaptation, CRISPR, Domestication, GWAS, Natural variation, Nitrogen limitation, *Saccharomyces cerevisiae*, Wild yeasts, Wine fermentation, Wine yeasts

## Abstract

For more than 20 years, *Saccharomyces cerevisiae* has served as a model organism for genetic studies and molecular biology, as well as a platform for biotechnology (e.g., wine production). One of the important ecological niches of this yeast that has been extensively studied is wine fermentation, a complex microbiological process in which *S. cerevisiae* faces various stresses such as limited availability of nitrogen. Nitrogen deficiencies in grape juice impair fermentation rate and yeast biomass production, leading to sluggish or stuck fermentations, resulting in considerable economic losses for the wine industry. In the present work, we took advantage of the “1002 Yeast Genomes Project” population, the most complete catalogue of the genetic variation in the species and a powerful resource for genotype-phenotype correlations, to study the adaptation to nitrogen limitation in wild and domesticated yeast strains in the context of wine fermentation. We found that wild and domesticated yeast strains have different adaptations to nitrogen limitation, corroborating their different evolutionary trajectories. Using a combination of state-of-the-art bioinformatic (GWAS) and molecular biology (CRISPR-Cas9) methodologies, we validated that *PNP1*, *RRT5* and *PDR12* are implicated in wine fermentation, where *RRT5* and *PDR12* are also involved in yeast adaptation to nitrogen limitation. In addition, we validated SNPs in these genes leading to differences in fermentative capacities and adaptation to nitrogen limitation. Altogether, the mapped genetic variants have potential applications for the genetic improvement of industrial yeast strains.

**Supplementary Information:**

The online version contains supplementary material available at 10.1186/s40659-023-00453-2.

## Background

*Saccharomyces cerevisiae* has been considered a central organism for over 20 years for genetics and molecular biology studies, being the first eukaryotic species with its genome completely sequenced [1]. Furthermore, this yeast is also a biological platform for biotechnology, with applications such as the production of bread, beer, wine, high-value metabolites, heterologous proteins, and vaccines [2–6]. This has led to the genome sequencing of a growing number of yeast strains, revealing the genomic characteristics that affect their adaptation to various natural and artificial niches [7, 8].

The first attempts to reveal yeast genetic diversity showed the presence of two yeast populations: domesticated yeasts associated with human activities (e.g., bread, beer and wine) and wild yeasts from natural environments (without human intervention) [9–11]. Recent genome sequencing studies have revealed population structure with greater precision [12–15], being the “1002 Yeast Genomes Project” the most complete catalogue of genetic variation in *S. cerevisiae* to date and a powerful resource for genotype-phenotype correlations in this species [16].

In the last decades, QTL (Quantitative Trait Loci) mapping has been the main experimental approximation to fill the gap between genotype and phenotype in yeast, being widely used to map the causatives genes that affect phenotypes such as thermotolerance, chemical resistance, translation termination, and production/consumption of metabolites during alcoholic fermentation, among many others [17–30]. However, other strategies like GWAS (Genome-Wide Association Studies), which has been successfully applied in human populations for the detection of disease-associated risk genetic variants, have rarely been used in yeast. This is due to the necessity of genotyping a large number of strains from diverse ecological niches, a problem that is completely overcome by the “1002 Yeast Genomes Project” population [16]. Therefore, GWAS seems an ideal next step to study the genetic bases of yeast adaptation to different natural and artificial niches.

An important ecological niche that has been extensively studied is the fermentative environment. *S. cerevisiae* is the main microorganism responsible for alcoholic fermentation in the winemaking process, contributing not only to the alcoholic degree but also to the flavours and aromas of the final product [31, 32]. Wine fermentation is a complex microbiological process where *S. cerevisiae* outperforms its competitors by transforming the sugars present in the grape must into ethanol [33] while facing various stresses, such as low pH (between 2 and 3), high osmotic pressure (20% of sugar concentration), high sulphite levels, ethanol toxicity, and limited nitrogen availability [34, 35].

Nitrogen limitation during wine fermentation is fundamental because, under this condition, yeast cells grow slowly, reducing ribosome biogenesis and protein translation, and arresting the cell cycle in G1 [36]. Nitrogen deficiencies in grape juice impair fermentation rate and yeast biomass production, leading to sluggish or stuck fermentations, resulting in considerable economic losses for the wine industry [37]. Recently, we have addressed the importance of natural diversity related to adaptation to low nitrogen levels, especially in terms of the recognition of wild yeast strains as a reservoir of beneficial alleles with potential industrial applications [38]. Therefore, performing a GWAS approach using the “1002 Yeast Genomes Project” population may allow us to search for wild alleles that favour the fermentation process and adaptation to nitrogen limitation, across the entire genetic diversity in *S. cerevisiae* described so far.

In the present work, we studied the adaptation to nitrogen limitation in wild and domesticated yeast strains belonging to the “1002 Yeast Genomes Project” population in the context of wine fermentation. To achieve this, we phenotyped this population under limited and non-limited nitrogen microfermentation conditions. We used this phenotypic information to (i) compare wild and domesticated yeast strains and (ii) perform GWAS analyses to map genetic variants underlying the studied phenotypes. We used a state-of the-art molecular biology tool, Clustered Regularly Interspaced Short Palindromic Repeats (CRISPR) associated with the Cas9 protein (CRISPR-Cas9) technique, to validate *RRT5*, *PDR12* and *PNP1* genes in the studied phenotypes and also to elucidate single nucleotide polymorphisms (SNPs) in these ORFs that lead to differential adaptation to nitrogen limitation. Overall, the identified alleles have potential applications for the genetic improvement of industrial wine yeast strains by modifying their genomes with specific point mutations, which could be an interesting approach for the wine industry.

## Results

### Comparing the adaptation of wild and domesticated yeast strains to nitrogen limitation

To evaluate the adaptation of a wide yeast population to a nitrogen-limited wine fermentation condition, we carried out microfermentations of the yeast strains belonging to the “1002 Yeast Genomes Project” [16]. These microfermentations were carried out in synthetic musts (SM) with limiting (SM60; 60 mg/L of yeast assimilable nitrogen (YAN)) and non-limiting (SM300; 300 mg/L of YAN) nitrogen contents, and we were able to collect data from 947 yeast strains that grew in both conditions (Additional file 1: Table S1).

To compare the adaptation of wild and domesticated yeasts, we first extracted four kinetic parameters from the growth curves: “efficiency” (proliferation efficiency), “rate” (proliferation rate), “lag” (proliferation lag) and “AUC (area under the curve)”. These parameters were obtained for each yeast strain in both growth conditions (SM60 and SM300). We calculated the ratio between the values obtained in SM60 and SM300 for each yeast strain as a specific measure of adaptation to nitrogen limitation (henceforth, “SM60/SM300 ratio” or simply “ratio”), obtaining a total of 12 different phenotypes (four kinetic parameters for the three conditions evaluated) (Additional file 1: Table S1). In addition, we classified each yeast strain in the population as “wild” (57 of 947), “domesticated” (560 of 947) or “unknown” (330 of 947), following previous criteria [39]; the “domesticated” category was further divided into “domesticated (wine)” (348 of 947) and “domesticated (non-wine)” (212 of 947), given if they belong to the “Wine/European” cluster or not [16] (Additional file 1: Table S1). Moreover, special attention was paid to the genomic information of each yeast strain in terms of ploidy, aneuploidies and heterozygosity levels (Additional file 1: Table S1).

**Fig. 1:**
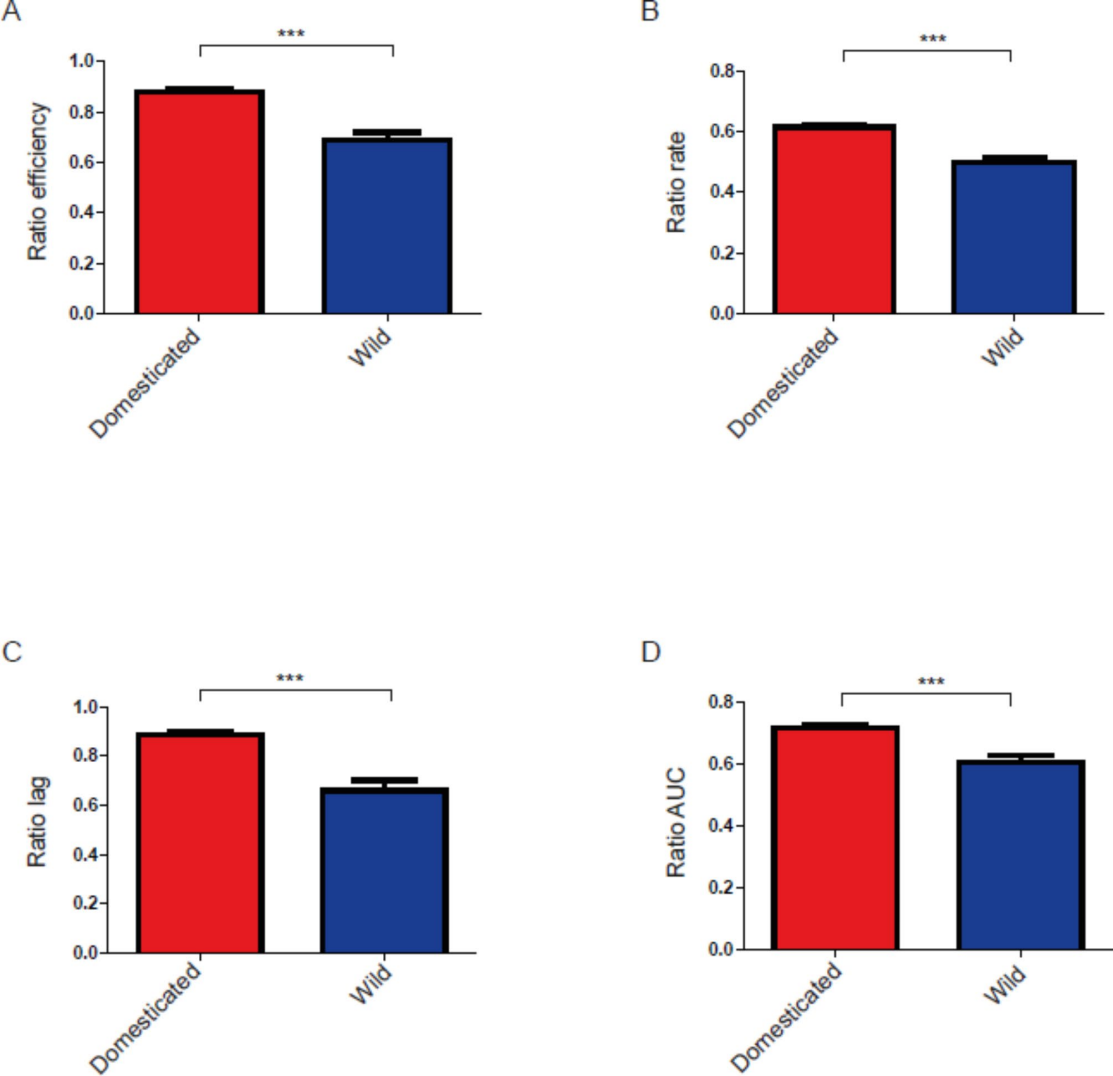
**Comparison between domesticated and wild strains for the SM60/SM300 ratio.** The kinetic parameters compared were (**A**) efficiency, (**B**) rate, (**C**) lag and (**D**) AUC. Statistical analyses correspond to two-tailed Mann Whitney tests. ***: p < 0.001, **: p < 0.01, *: p < 0.05

The results showed that, although in SM300 there were almost no differences between domesticated and wild yeast strains (except in terms of rate and efficiency) (Additional file 2: Figure S1), in SM60 there were clear differences between these two groups, with the domesticated yeast strains having higher values for all the four parameters under study (Additional file 2: Figure S2). This led to the higher values of the four parameters for domesticated yeast strains when considering the SM60/SM300 ratio, indicating that these two groups have different adaptations to the nitrogen-limited condition: while domesticated yeast strains have higher efficiency, rate and AUC values, wild yeast strains have lower lag values (Fig. 1).

**Fig. 2:**
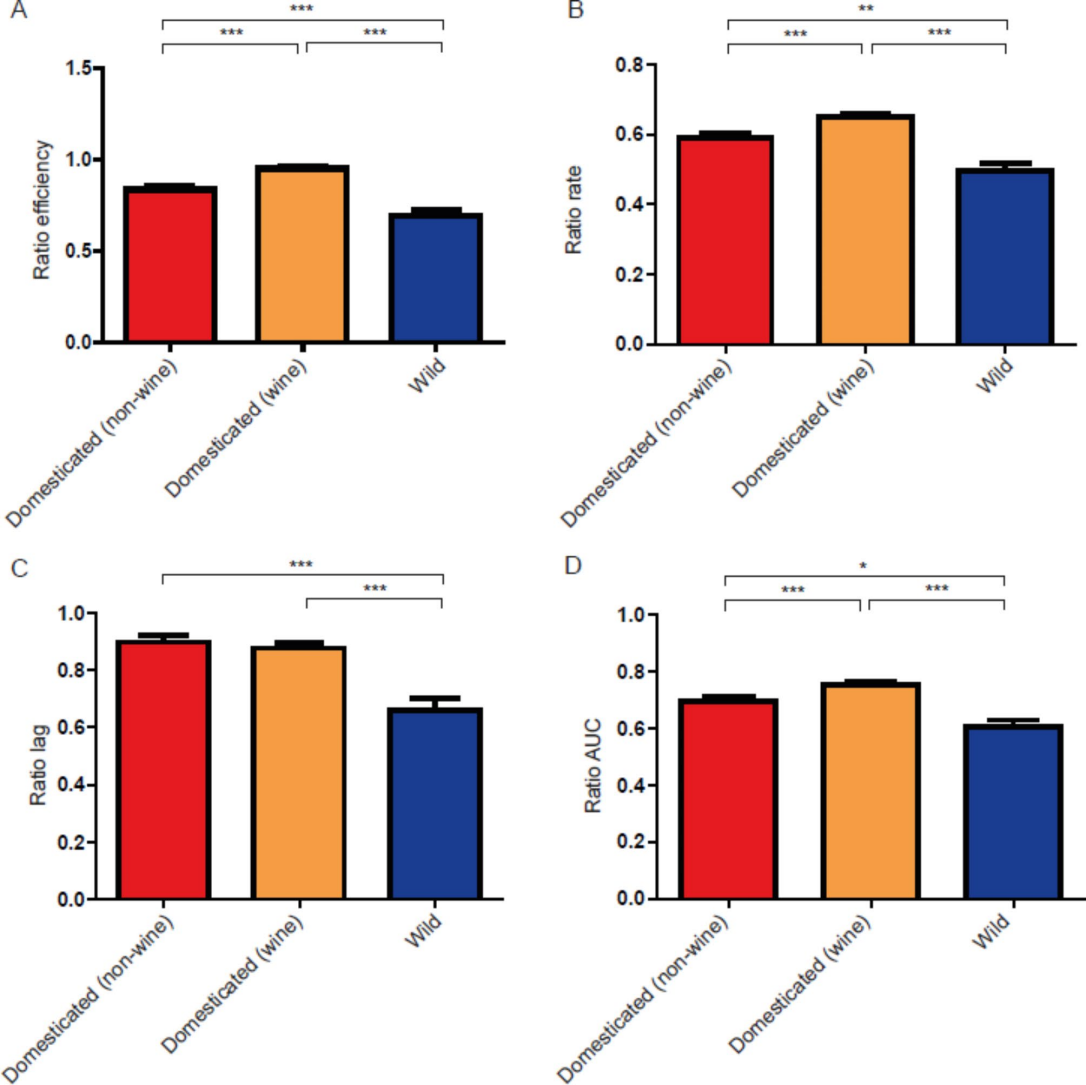
**Comparison between domesticated (non-wine), domesticated (wine) and wild strains for the SM60/SM300 ratio.** The kinetic parameters compared were (**A**) efficiency, (**B**) rate, (**C**) lag and (**D**) AUC. Statistical analyses correspond to Kruskal-Wallis tests using Dunn’s multiple comparisons tests. ***: p < 0.001, **: p < 0.01, *: p < 0.05

Next, we analysed the phenotypes separating the wine and non-wine domesticated yeast strains, observing a similar scenario. For instance, while neither in SM300 nor in SM60 the wine domesticated yeast strains had the highest rate value (Additional file 2: Figures S3-S4), they had the highest value in terms of the SM60/SM300 ratio for this parameter, same for efficiency and AUC values (Fig. 2). Furthermore, wild yeast strains showed the same pattern compared to the separated wine and non-wine domesticated yeast strains, with lower values of all four kinetic parameters compared to both domesticated groups (Fig. 2).

Genomic features such as ploidy and aneuploidies could partly explain the observed phenotypic differences. To confirm this, we compared the values obtained for the four parameters under study in terms of ploidy (haploids vs. diploids vs. polyploids) and aneuploidies (euploids vs. aneuploids), observing some differences between these groups, although not for all the kinetic parameters and conditions evaluated (Additional file 2: Figures S5–S10). Particularly interesting is that, while no differences were observed in the SM60/SM300 ratios of the four parameters when comparing ploidy levels, when considering aneuploidies, all four SM60/SM300 ratios showed differences (Additional file 2: Figures S7–S10). When we repeated the comparisons between the wild, wine domesticated and non-wine domesticated groups, but considering only the diploid-euploid yeast strains of each group, we obtained the same results as before (Additional file 2: Figures S11–S13).

### Mapping genetic variants involved in yeast adaptation to nitrogen limitation

**Fig. 3:**
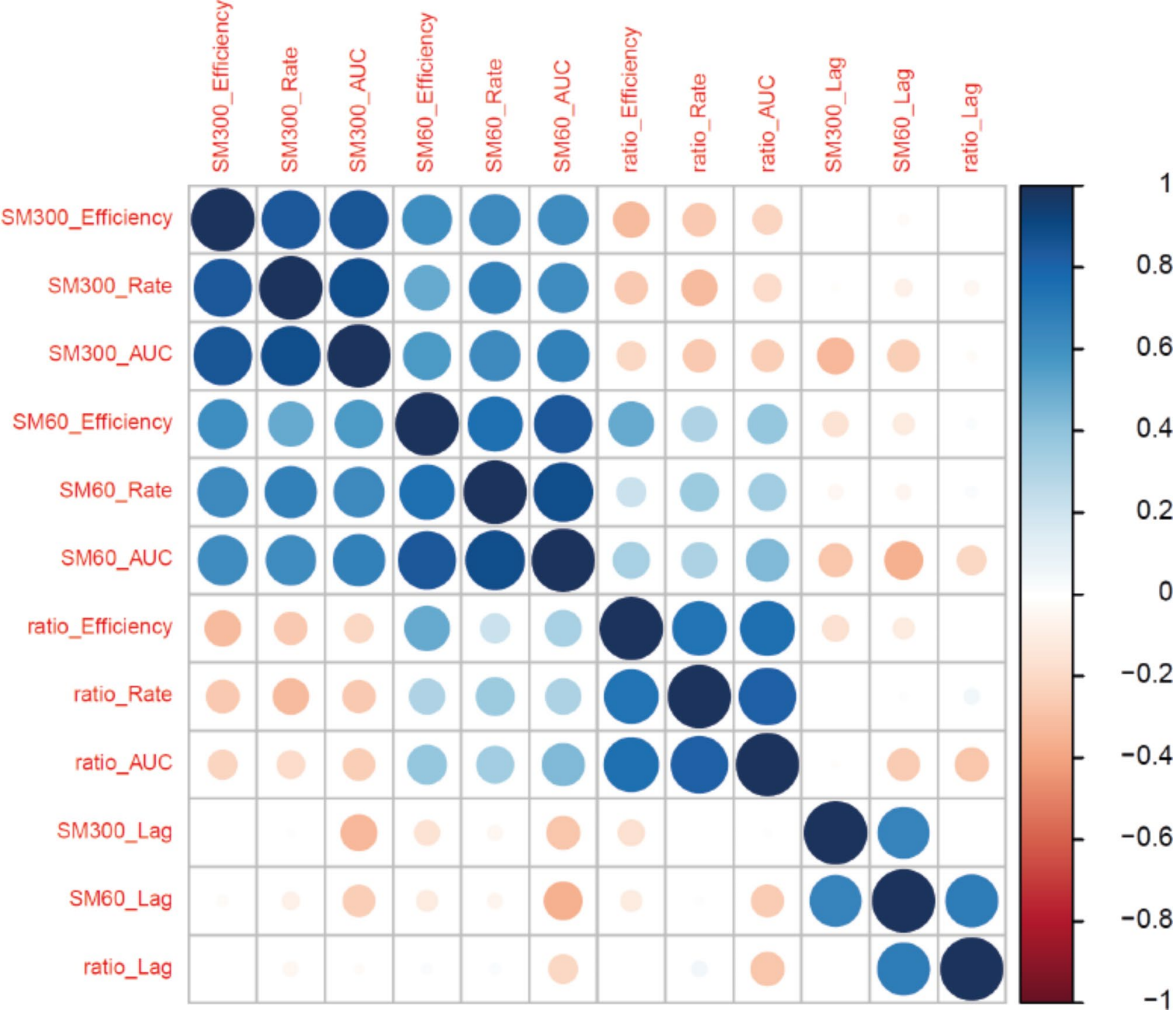
**Correlations between the phenotypes studied by GWAS.** The colour scale goes from total negative correlation (red) to total positive correlation (blue), going through no correlation (white). The diameter of each circle is proportional to the colour scale to better highlight the correlations obtained

To identify the causative variants associated with the nitrogen-limited microfermentations, we performed the GWAS analyses on all the 12 phenotypes previously mentioned. We considered only the diploid-euploid yeast strains (594 yeast strains in total) to have more reliable results, since both aneuploidies and ploidy (haploidy and polyploidy) may have large confounding effects when included in the GWAS [39]. From these analyses, we were able to map 109 different genetic variants, including loss-of-function (LOF) (1 of 109), copy number variants (CNVs) (13 of 109), and SNPs in both coding and non-coding regions (95 of 109), with some of these variants (19 of 109) being pleiotropic (i.e., affecting more than one phenotype) (Additional file 1: Table S2 and Additional file 2: Figures S14–S16). We also searched for correlations among the phenotypes under study. In general, we found a positive correlation among the efficiency, rate and AUC parameters, and a lack of correlation (or even a negative correlation) between these parameters and the lag (Fig. 3).

We further analysed the genetic variants obtained, looking for interesting candidates to be experimentally validated. We considered criteria such as variants having a low p-value, pleiotropic variants, genes with more than one variant affecting the same phenotype, and variants occurring in non-essential genes (according to the *Saccharomyces* genome database (SGD)). We finally selected three genes and their associated genetic variants for further validation: *PNP1*, which encode a purine nucleoside phosphorylase and carries a SNP affecting three phenotypes in SM300 (efficiency, rate and AUC); *RRT5*, which encode a putative protein of unknown function and contains two SNPs in its coding region affecting the SM60/SM300 ratio of AUC; and *PDR12*, which encode a plasma membrane ATP-binding cassette (ABC) transporter and includes three SNPs (two in the coding region and one in the regulatory region) affecting SM60/SM300 ratio of lag (Table 1).

**Table 1:** Genetic variants mapped by GWAS and selected for validation

Gene	Gene ID	Position ^a^	Reference SNP ^a^	Mutant SNP ^b^	Phenotype
*PNP1*	YLR209C	933	A	G	SM300_AUC SM300_Efficiency SM300_Rate
*RRT5*	YFR032C	579	C	T	Ratio_AUC
		601	A	C	
*PDR12*	YPL058C	2781	T	C	Ratio_Lag
		4393	G	A	

^a^ Corresponding to the allele present in the reference yeast genome of the SGD (S288c strain) [80]. ^b^ Corresponding to the alternative allele present in the population

### Specific SNPs account for differential adaptation to nitrogen limitation

**Fig. 4:**
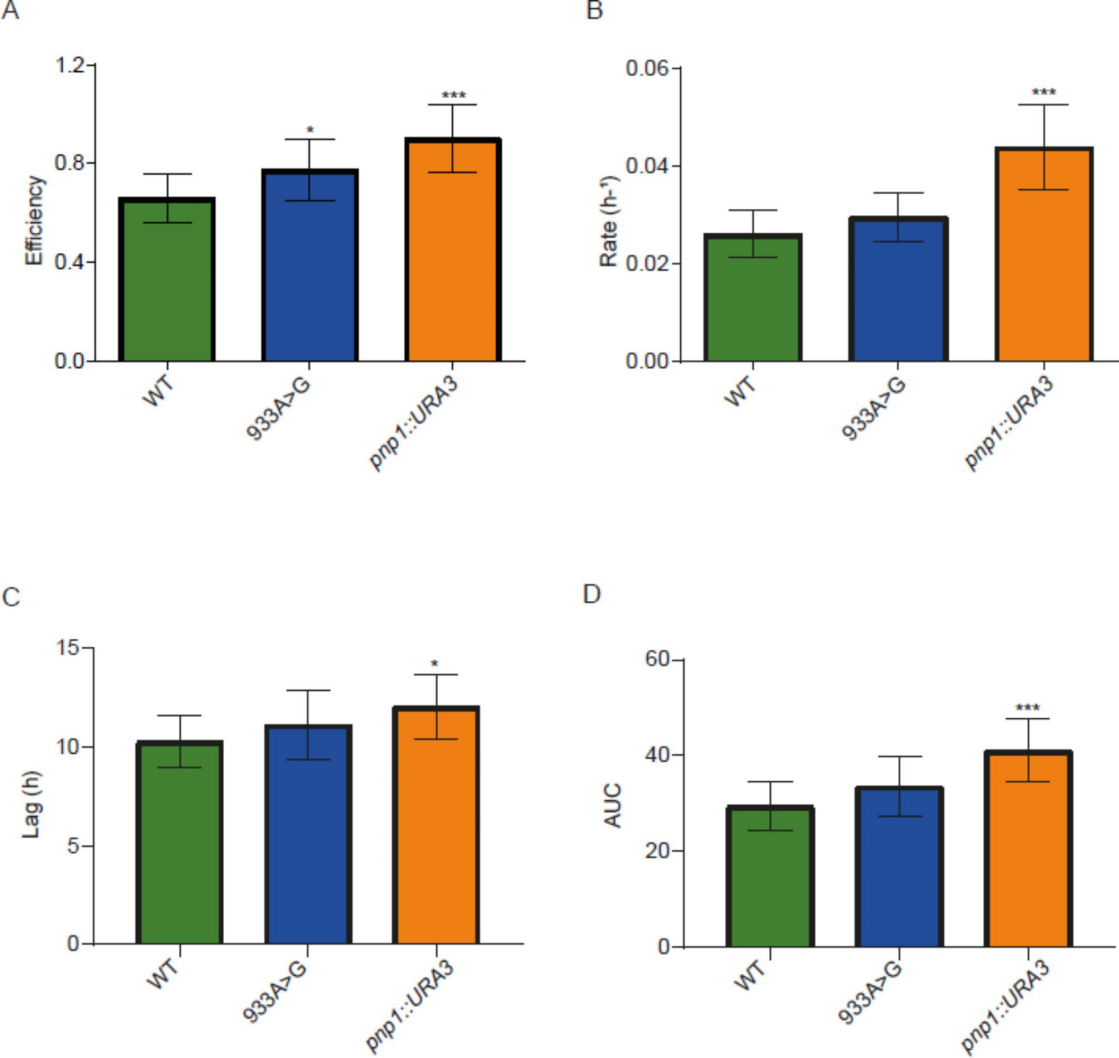
**Comparison between *PNP1 *mutants and their wild type (WT) strain in SM300.** The kinetic parameters compared were (**A**) efficiency, (**B**) rate, (**C**) lag and (**D**) AUC. Statistical analyses correspond to ordinary one-way ANOVA using Holm-Šídák’s multiple comparisons tests, comparing in each case the WT versus the different mutants. ***: p < 0.001, **: p < 0.01, *: p < 0.05

**Fig. 5:**
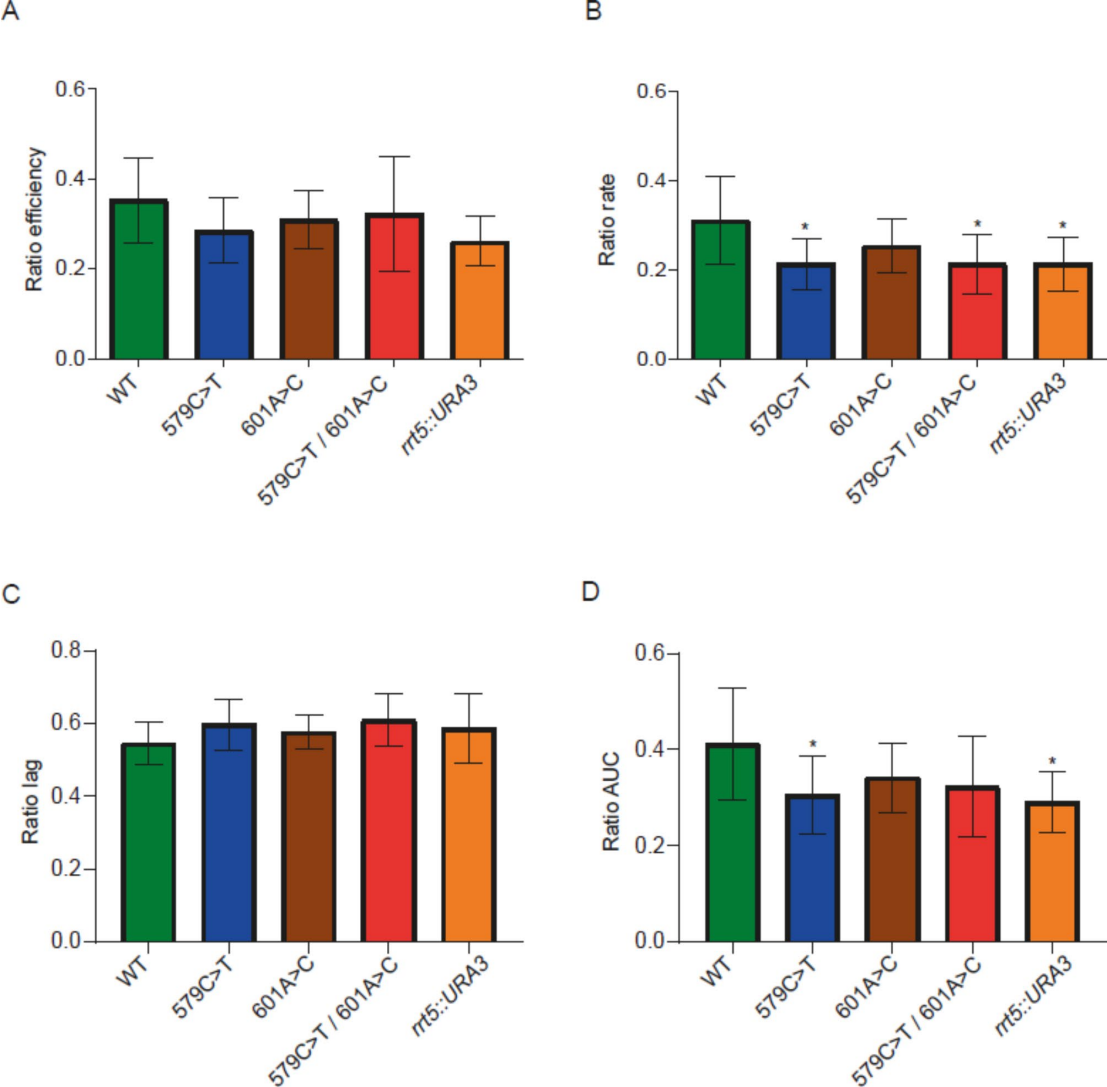
**Comparison between *RRT5 *mutants and their wild type (WT) strain for the SM60/SM300 ratio.** The kinetic parameters compared were (**A**) efficiency, (**B**) rate, (**C**) lag and (**D**) AUC. Statistical analyses correspond to ordinary one-way ANOVA using Holm-Šídák’s multiple comparisons tests, comparing in each case the WT versus the different mutants. ***: p < 0.001, **: p < 0.01, *: p < 0.05

**Fig. 6:**
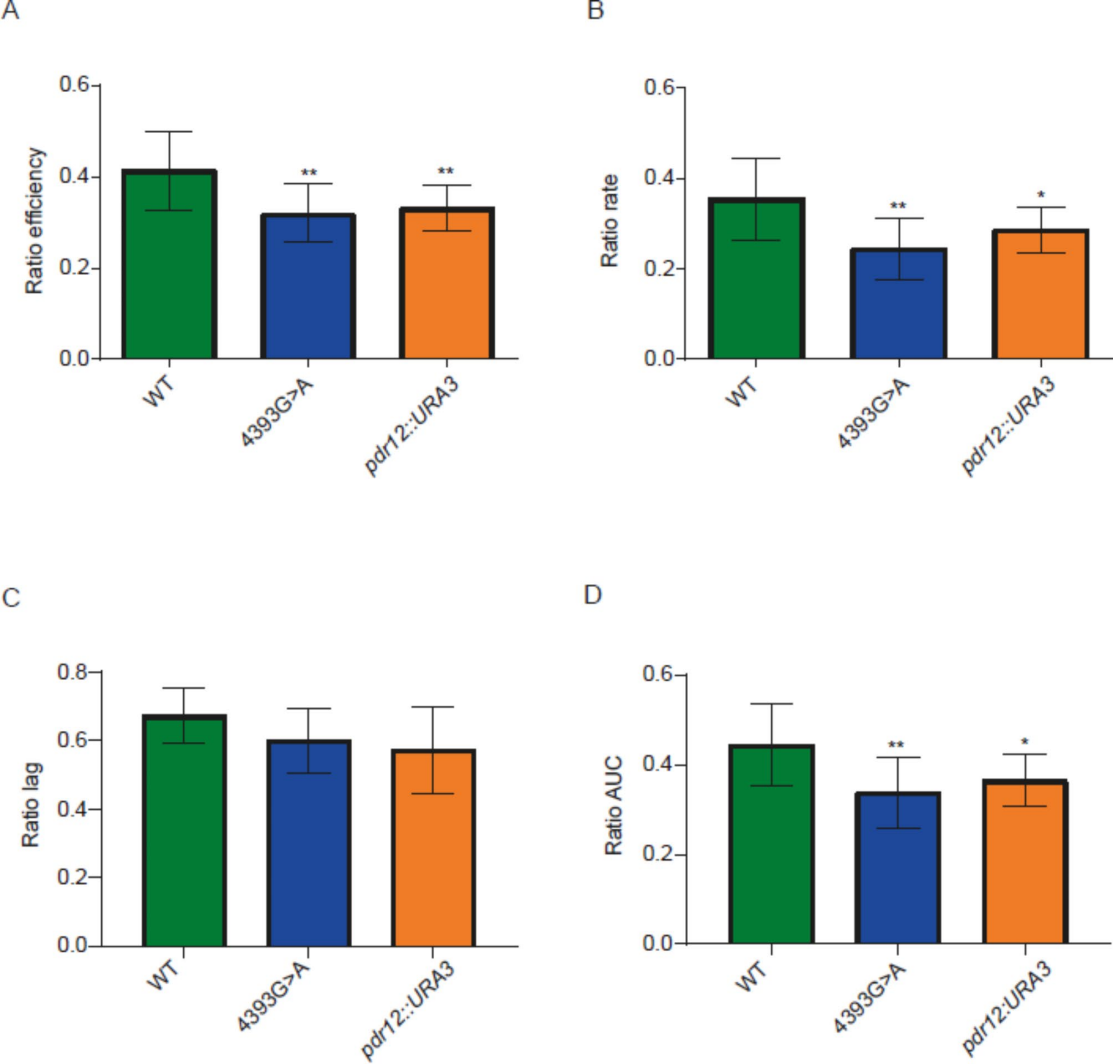
**Comparison between *PDR12 *mutants and their wild type (WT) strain for the SM60/SM300 ratio.** The kinetic parameters compared were (**A**) efficiency, (**B**) rate, (**C**) lag and (**D**) AUC. Statistical analyses correspond to ordinary one-way ANOVA using Holm-Šídák’s multiple comparisons tests, comparing in each case the WT versus the different mutants. ***: p < 0.001, **: p < 0.01, *: p < 0.05

We validated the involvement of some of the genetic variants identified by GWAS at two levels: whole gene level and SNP level. First, to validate the involvement of the previously selected genes in the studied phenotypes, we generated null mutants of these genes in a haploid laboratory genetic background (BY4741 strain). Then, we evaluated these mutants in the same microfermentation conditions used previously. The results indicate that the three selected genes are involved in some of the studied phenotypes (Figs. 4, 5 and 6 and Additional file 1: Table S5). *PNP1* null mutant showed the expected differences in efficiency, rate and AUC in SM300, but also in lag (Fig. 4). On the other hand, *RRT5* null mutant showed the expected differences in AUC in SM60/SM300 ratio, but also in rate (Fig. 5); the same pattern was observed in SM60, but not in SM300 (Additional file 2: Figures S17–S18). Finally, *PDR12* null mutant did not show the expected differences in lag in SM60/SM300 ratio (although it had a p-value very close to the statistical cut-off (0.0641)) but did for the other three parameters (Fig. 6). However, statistically significant differences in lag were observed in both SM300 and SM60 (Additional file 2: Figures S19–S20).

We then generated allelic mutants that carry only the SNPs mapped by GWAS to validate the involvement of these genetic variants in the studied phenotypes. In the case of *PNP1*, we found a yeast strain (named “SACE-YCP”) in the “1002 Yeast Genomes Project” population that has an allele that differs from the BY4741 allele only at the desired position (933A>G), so we performed the allele swapping of this allele into the BY4741 strain. This corresponds to a synonymous mutation of a leucine in the amino acid sequence of Pnp1 (Table 2). Regarding the observed phenotypes, although we did not observe differences in rate, lag and AUC in SM300 as in the case of the null mutant, we validated that this SNP is causing a difference in growth efficiency in SM300, confirming its involvement in this phenotype (Fig. 4).

**Table 2:** Amino acid changes caused by the mapped genetic variants

Protein	Position ^a^	Type of mutation	Reference AA ^a^	Mutant AA ^b^
Pnp1	311	Synonymous	Leu (L)	Leu (L)
Rrt5	193	Synonymous	Thr (T)	Thr (T)
	201	Missense (non-conservative)	Ser (S)	Arg (R)
Pdr12	927	Synonymous	Asn (N)	Asn (N)
	1465	Missense (conservative)	Val (V)	Ile (I)

^a^ Corresponding to the allele present in the reference yeast genome of the SGD (S288c strain) [80]. ^b^ Corresponding to the alternative allele present in the population. AA: Amino acid

In the cases of *RRT5* and *PDR12*, we could not find yeast strains in the population carrying alleles with just the SNPs needed, so we had to introduce them with CRISPR-Cas9 technology. This was technically difficult because the target region must include the SNP to be changed, limiting the region for gRNA design, which is key to correctly applying this technique [40]. This leads to the fact that, in the case of *PDR12*, only one of the two desired point mutations could be made (4393G>A), which corresponds to a conservative missense mutation from valine to isoleucine (V1465I) in the amino acid sequence of Pdr12 (Table 2). However, both desired point mutations were made for *RRT5* (579C>T or 601A>C), including an allele carrying both point mutations (579C>T and 601A>C); these changes correspond to a synonymous mutation of threonine in the amino acid sequence of Rrt5, and to a non-conservative missense mutation from serine to arginine (S201R), respectively (Table 2).

Regarding the observed phenotypes, one of the *RRT5* allele mutants carrying only one point mutation (579C>T) showed phenotypic differences in rate and AUC in SM60/SM300 with respect to the WT strain; in contrast, the allele mutant carrying the other point mutation (601A>C) showed no differences in the parameters assayed (Fig. 5). In addition, the allele mutant carrying the 579C>T point mutation showed the same pattern as the null mutant for the SM60/SM300 ratios of the four analysed parameters (Fig. 5). Interestingly, the allele mutant carrying both mutations (579C>T and 601A>C) showed an intermediate behaviour, with differences in rate but not in AUC (Fig. 5). None of the *RRT5* allele mutants showed the same profile as the null mutant in either SM300 or SM60 separately (Additional file 2: Figures S17–S18). On the other hand, the *PDR12* allele mutant (4393G>A) showed the same profile of phenotypic differences as its null mutant, showing differences in efficiency, rate and AUC in SM60/SM300, but not in lag (Fig. 6); the same pattern was observed in SM60, but not in SM300 (Additional file 2: Figures S19–S20). Overall, these results demonstrate not only the involvement of *PNP1*, *RRT5* and *PDR12* in the studied phenotypes but also that the SNPs in these genes lead to differences in growth kinetics in wine fermentation conditions, with a direct impact on the differential adaptation to nitrogen limitation.

## Discussion

In recent decades, the natural variation of *S. cerevisiae* has been massively exploited to understand ecological and evolutionary processes. This has resulted in the discovery of genetic variants underlying complex traits that represent a rich genetic resource with the potential to provide new yeast strains for industrial fermentation processes [41]. In this context, wild yeast strains are particularly interesting to study, as they harbour higher genetic and phenotypic diversity than industrial (domesticated) yeast strains and are potentially a reservoir of beneficial alleles for genetic improvement [38, 42]. In the present work, we took advantage of the “1002 Yeast Genomes Project” population [16], the most powerful genetic resource to date, to study the adaptation to nitrogen limitation in the context of wine fermentation.

A widely used strategy for the phenotyping of a large number of yeast strains is to perform microculture experiments and obtain kinetic parameters from the growth curves, such as efficiency of proliferation, rate of proliferation and lag of proliferation [43]. Previous studies have found a strong correlation between efficiency and rate but no correlation (or even a negative) between lag and the other two parameters [23, 44]. Furthermore, the existing correlation between efficiency and rate depends largely on the environment [44] instead of biomass yield limitation as has been suggested in other works [45]. The present results corroborate these observations (Fig. 3), consistent with reports on artificial LOF mutants in *S. cerevisiae* [46], refuting the hypothesis of an evolutionary trade-off between efficiency and rate [47]. This suggests that efficiency and rate have similar underlying genetic structures, while being physiologically and evolutionarily distinct from lag [44].

We decided to add a fourth parameter in this study, AUC, as a more comprehensive parameter that could integrate the other three. The results show that this parameter positively correlates with efficiency and rate (Fig. 3), indicating that these parameters contribute more to AUC than lag. This is also corroborated by GWAS results, e.g., with the genetic variant of *PNP1* gene that was mapped for efficiency, rate and AUC in SM300 (Table 1 and Additional file 1: Table S2). However, genetic variants were also mapped exclusively for AUC, e.g., for the *RRT5* gene (Table 1 and Additional file 1: Table S2). These results indicate that, strongly correlated with efficiency and rate, AUC could provide new information on yeast adaptation when studying growth curves. To be used routinely by the yeast research community, a consensus is needed on the best way to calculate this parameter so that it can be automatically estimated by an equation such as the Gompertz growth equation [48].

Regarding the comparison between wild and domesticated yeast strains, the results show that these two groups of yeasts have different adaptions to nitrogen limitation, measured as the ratio between the values of the parameters studied in SM60 and SM300. In principle, it can be expected that domesticated yeast strains are better adapted than wild ones to nitrogen limitation due to their use in that condition. However, recent evidence points in the opposite direction, given the scarcity of nitrogen sources that wild yeast strains have to face in natural environments, and therefore the need for these strains to adapt to this limitation [30, 38]. The results show that domesticated yeast strains have higher values of efficiency, rate and AUC, but, interestingly, wild yeast strains show a lower value of lag (Figs. 1 and 2). In terms of an industrial process like wine fermentation, what a winemaker wants is to have a yeast strain that shows high efficiency and high rate, but low lag. Since wild yeast strains show a lower lag compared to the domesticated yeast strains, they could act as reservoir of beneficial alleles with potential industrial applications, particularly in terms of decreasing the lag phase in response to nitrogen limitation. These results reinforce the idea that wild and domesticated yeast populations reflect different evolutionary trajectories [9–11, 49, 50].

Using the parameters obtained from the microculture growth curves as phenotypic information, we mapped 109 genetic variants by GWAS (Additional file 1: Table S2 and Additional file 2: Figures S14–S16). Interestingly, several genetic variants are found in the coding and/or regulatory regions of genes mapped for adaptation to nitrogen limitation in a previous work of our group [30]: *USE1* and *ECM38*, which were previously identified by QTL mapping; *MHO1*, which showed *de novo* mutations; *RRT5*, *OPT2*, *ECM38*, *ADY3* and *CDA1*, whose transcription was upregulated in SM60 compared to SM300; and *ATR1* and *RPL42A*, whose transcription was downregulated in SM60 compared to SM300. In the present work, we found several genetic variants that fall within autonomously replicating sequences (ARSs) (sv9386, sv43187, sv57282, sv65176, sv73285, sv73286, sv78694), or close to an ARS (sv50798) or a tRNA (sv56522, sv56526). However, a detailed study is needed to find a direct relationship between ARSs/tRNAs and wine fermentation. In the case of tRNAs, this relationship could be mediated by the TORC1 signalling pathway [24].

Another interesting identified genetic variant is the LOF in the *SSK22* gene. LOFs in the population are caused by a variety of mutations, clustered together; these mutations are those predicted to have high impact by SnpEff [51]. In the *SSK22* gene, we found 13 possible mutations causing potential LOF: one possible starting codon loss (3G>A) and 12 different nonsense mutations (181C>T, 434G>A, 673G>T, 1065T>G, 1294C>T, 1747C>T, 1781T>A, 1900C>T, 2701C>T, 3289C>T, 3406C>T and 3964G>T). *SSK22* encodes a MAP kinase kinase kinase of *HOG1* mitogen-activated signalling pathway [52, 53], whose LOF SNPs are candidates for validation in future work.

We investigated in more detail three genes that harbour some of the genetic variants mapped by GWAS (Table 1). We validated their involvement in wine fermentation (*PNP1*) (Fig. 4) and adaptation to nitrogen limitation (*RRT5* and *PDR12*) (Figs. 5 and 6), using the BY4741 laboratory strain as genetic background. Although this strain has lower fermentation capacities compared to an industrial strain, leading to large error bars for the kinetic parameters (Figs. 4, 5 and 6), the observed phenotypic differences between isogenic strains, among which the only genetic variation is a unique SNP, allowed us to associate these genetic variants with the phenotypes under study. An interesting approach would be to introduce these same mutations into an industrial strain and perform not only microculture experiments but also larger-scale fermentations, which would allow us to assess both the effect of the industrial genetic background and the impact of the SNPs on other fermentative phenotypes, such as nitrogen consumption and ethanol production. It is important to highlight that, to the best of our knowledge, the present work provides the first evidence of the participation of *PNP1* and *RRT5* in wine fermentation and confirms the participation of *PDR12* in this process.

The first of them, *PNP1*, encodes a purine nucleoside phosphorylase that specifically metabolizes inosine and guanosine nucleosides, being involved in the nicotinamide riboside salvage pathway [54, 55]. It has been shown that Pnp1 is associated with the control of ATP homeostasis during the respiro-fermentative transition in yeast: under glucose-depleted conditions, this protein regulates the accumulation of inosine, restoring ATP levels [56]. Furthermore, its hemizygous mutant showed lower relative growth compared to its WT control (S288c background) in fermentation production medium (FPM) (i.e., showed haploinsufficiency) [57]. These antecedents support the phenotypic changes observed in the null mutant and the allele mutant of this gene, for efficiency, rate and AUC in SM300 (Fig. 4).

On the other hand, *RRT5* is a non-essential gene identified in a screen for mutants with increased levels of rDNA transcription, which encodes a putative protein of unknown function; it is also highly expressed during sporulation [58, 59]. As in the case of *PNP1*, its hemizygous mutant showed lower relative growth compared to its WT control in FPM [57]. Moreover, its deletion caused a decreased rate of glutamine utilization as a nitrogen source [60], which is an antecedent that reinforces the idea that this gene is involved in the adaptation to nitrogen limitation. This can be seen for rate and AUC for the SM60/SM300 ratio in the null mutant and in at least one of the two SNPs tested (Fig. 5). In addition, its deletion also caused a defect in vacuolar fragmentation, which could be related to nitrogen utilization through the TORC1 complex, which is located in the vacuolar membrane [24].

Finally, *PDR12* encodes a plasma membrane ABC transporter, more specifically a weak-acid-inducible multidrug transporter required for weak organic acid resistance [61–64]. For example, Pdr12 is involved in the export of fusel acids derived from amino acids such as leucine, isoleucine, valine, phenylalanine and tryptophan, linking the nitrogen source with the expression of this transporter [65]. Regarding the fermentation process, a hemizygous yeast strain for *PDR12* showed haploproficiency in FPM [57]; moreover, this gene was found to be differentially expressed in phases I (exponential) and II (stationary) of a batch wine fermentation [66]. This evidence supports that *PDR12* is implicated in yeast adaptation to nitrogen limitation, due to the phenotypic changes caused by both the null mutant and the allele mutant of this gene, for efficiency, rate and AUC in the SM60/SM300 ratio (Fig. 6).

Interestingly, out of the four evaluated SNPs, two correspond to synonymous mutations, one to a conservative missense mutation, and only one to a non-conservative missense mutation (Table 3). Although it is generally accepted that missense mutations (especially the non-conservative ones) could have greater phenotypic effects by directly impacting protein function, it has been well established that synonymous mutations can also have phenotypic effects, potentially impacting translation efficiency (in terms of elongation rate and/or accuracy) [67], and even gene expression levels [68]. In yeast, it has been suggested that transcriptional mechanisms may play a role in shaping codon bias, with mRNA secondary structure connecting transcriptional activity to codon bias in highly expressed genes [69]. In terms of codon usage, while the silent mutation in *PNP1* changes the WT codon (TTA) for another (TTG) with almost the same frequency (26.32 vs. 26.48 per thousand, respectively), the silent mutation in *RRT5* changes the WT codon (ACT) for another (ACC) with lower frequency (20.22 vs. 12.47 per thousand, respectively) [70], which could explain the phenotypic differences observed (Fig. 5 and Additional file 2: Figures S17–S18).

**Table 3:** Yeast strains used in the present work for validations

Yeast strain	Relevant genotype	Source
BY4741	*MATa*; *his3∆1*; *leu2∆0*; *met15∆0*; *ura3∆0*	Lab strain
SACE-YCP	-	(16)
Y184	BY4741; *pnp1::URA3*	This work
Y223	BY4741; *PNP1*: g.933A>G	This work
YC546	BY4741; *RRT5*: g.579C>T	This work
YC548	BY4741; *RRT5*: g.601A>C	This work
YC549	BY4741; *RRT5*: g.579C>T,g.601A>C	This work
YC552	BY4741; *rrt5::URA3*	This work
YC571	BY4741; *pdr12::URA3*	This work
YC572	BY4741; *PDR12*: g.4393G>A	This work

The results obtained show the power of the combination of GWAS and CRISPR to elucidate the genetic bases of a complex trait, such as adaptation to nitrogen limitation, at the level of single nucleotide resolution. Although we did not directly link the validated SNPs with a “wild” origin, the results obtained has clear applied potential to improve an industrial wine yeast strain by introducing the validated SNPs using CRISPR, which could be an interesting approach for the wine industry. It is worth mentioning that genetic engineering has rarely been used for yeast improvement in the food industry, mainly due to legal restrictions and consumer rejection; however, new technologies collectively known as “new breeding techniques” (NBTs) are challenging this paradigm, and CRISPR has been used experimentally to produce a wide variety of commercial genetically modified (GM) crops, such as maize and soybean [71].

## Conclusions

In the present work, we studied the adaptation to nitrogen limitation in the context of wine fermentation using the “1002 Yeast Genomes Project” population, which is the most complete catalogue of the genetic variation in *S. cerevisiae* to date. By comparing different kinetic parameters of growth curves obtained in microculture experiments, we found that wild and domesticated yeast strains have different adaptations to nitrogen limitation, corroborating that these two groups have different evolutionary trajectories. Using a combination of state-of-the-art bioinformatic (GWAS) and molecular biology (CRISPR) methodologies, we validated that *PNP1*, *RRT5* and *PDR12* are implicated in wine fermentation, where *RRT5* and *PDR12* are also involved in yeast adaptation to nitrogen limitation. Moreover, we validated that SNPs in these genes lead to differences in the studied phenotypes. Therefore, the genetic variants mapped have potential applications for the genetic improvement of industrial yeast strains for the wine industry.

## Methods

### Yeast strains

*“1002 Yeast Genomes Project” population*.

In the phenotyping experiments, we used 974 of the 1011 fully sequenced yeast strains belonging to the “1002 Yeast Genomes Project” [16]. For GWAS experiments, we considered the phenotypic data of a subset of this population, which consisted solely of diploid-euploid (594 yeast strains in total). All the yeast strains used are listed in Additional file 1: Table S1.

#### Null mutants and allelic mutants

To validate the involvement of the genetic variants identified from GWAS experiments, we generated a set of mutant yeast strains from the BY4741 laboratory strain. Two types of mutants were generated: “null mutants”, in which the entire ORF under study was replaced by a selection marker (*URA3* gene); and “allelic mutants”, in which we obtained yeast strains with only the point mutation under study for each selected ORF, either through allele swapping or CRISPR-Cas9 techniques (see below for more details). All the yeast strains generated in the present work are listed in Table 3.

### Phenotyping of the “1002 yeast genomes Project” population

#### Synthetic must composition

The experiments were carried out with synthetic musts that mimic natural grape musts but with defined compositions, prepared as previously described [23]. The composition of sugar was 250 g/L in total (125 g/L glucose and 125 g/L fructose), while the composition of nitrogen sources was 40% ammonium and 60% amino acids. The concentration of nitrogen sources was modified at two different levels: 60 mg/L (SM60) and 300 mg/L (SM300) of YAN, which correspond to limiting and non-limiting nitrogen conditions, respectively [24, 72].

### Microculture fermentations

Yeast strains were phenotyped under fermentative microculture conditions (SM300 and SM60) by monitoring the OD_600_ of the cells using 30 min intervals on a Tecan Sunrise microplate reader (Tecan, Germany). The relative fitness variables (growth parameters) for each yeast strain were calculated as previously described [73]. Briefly, proliferation efficiency (“efficiency”), proliferation rate (“rate”) and proliferation lag time (“lag”) were extracted from high-density growth curves using Gompertz growth Eq. [48] in Graph Pad Prism 7.04 software. In addition, we calculated the area under the growth curve (AUC) as a fourth growth parameter. Statistical analyses of these parameters consisted of one-way ANOVA using Holm-Šídák’s multiple comparisons test, which were also performed using Graph Pad Prism 7.04 software. All microculture experiments were carried out in three independent biological replicas for the “1002 Yeast Genomes Project” population and twelve independent biological replicas for the null mutants and allelic mutants of the BY4741 strain.

### Genome-wide association studies

GWAS experiments were performed as previously described [16, 39]. Briefly, we used the previously obtained phenotypic information and the genotypic information of the yeast strains belonging to the “1002 Yeast Genomes Project” to run the FastLmmC program, which implements the “Factored Spectrally Transformed Linear Mixed Model” (FaST-LMM) algorithm and considers the stratification of the lineages present in the yeast population [74]. We took into consideration only the diploid-euploid yeast strains from the population. SNPs, LOF, gene presence/absence, and CNVs matrices used in GWAS were taken from [39]. In the variant matrix, SNPs with a minor allele frequency (MAF) greater than 5% in the population were included along with the most frequent minor allele for non-biallelic SNPs. We used the SNPs matrix with MAF > 0.5% in GWAS to correct for population structure. We used a threshold of 5% family-wise error rate for p-values in GWAS to determine whether markers are significantly associated with phenotypes. The alleles mapped by GWAS were further analysed using the SGD (https://www.yeastgenome.org/) [75] to confirm their association with the studied phenotypes.

### Generation of mutant yeast strains

#### Transformation method

Yeast transformations and co-transformations were carried out using the standard lithium acetate transformation protocol [76] in the BY4741 genetic background (*MATa*; *his3∆1*; *leu2∆0*; *met15∆0*; *ura3∆0*). All the PCR amplifications were performed using the Phusion Flash High Fidelity Master mix (Thermo Scientific, USA) according to the manufacturer’s instruction. Primers and gRNAs used for cloning, deletion, allele replacement and CRISPR-Cas9 are listed in Additional file 1: Table S3–S4. The yeast strains used for validations in this work are listed in Table 3.

### Null mutants of *PNP1*, *RRT5* and *PDR12* genes

Null mutants for the three genes under study were generated by PCR amplification of the *URA3* gene and direct replacement by homologous recombination at each locus. Deletions were carried out using primers with 50 bp of overhang for direct recombination with the yeast genome and confirmed by standard colony PCR using primers upstream and downstream of each locus.

### Allelic mutants of *PNP1* gene (allele swapping)

Allele swapping of *PNP1* in the BY4741 genetic background was carried out according to a previously described method [77]. Briefly, the *PNP1* allele from the SACE-YCP strain, which belongs to the “1002 Yeast Genomes Project” [16], was amplified by PCR using genomic DNA. The *PNP1* allele included 936 bp from the coding sequence plus 300 bp of the transcriptional terminator. The *PNP1* allele was fused with the *HphMx* cassette downstream of the *PNP1* terminator and cloned into the pRS426 plasmid using yeast recombinational cloning, and using PCR primers with 50 bp of overhang between adjacent PCR products for homologous recombination [78]. The SACE-YCP *PNP1* allele was then amplified by PCR from pRS426 plasmid, and the PCR product was used to transform the previously generated BY4741 *PNP1∆* strain, replacing the *URA3* gene with the SACE-YCP *PNP1* allele. The correct replacement of the *URA3* gene by the SACE-YCP *PNP1* allele was confirmed by PCR amplification and sequencing of the PCR product (Macrogen, Republic of Korea).

### Allelic mutants of *RRT5* and *PDR12* (CRISPR-Cas9)

gRNAs design involved a short 20 nucleotides sequence with the PAM sequence (5’-NGG-3’) at their 3’ end. For specific nucleotide changes, gRNAs sequences were designed at the position of interest (579 and 601 for *RRT5* gene, and 4393 for *PDR12* gene) using Benchling software (https://www.benchling.com/), with the genomic sequence of S288c strain as reference. The designed primers were cloned in the pAEF5 plasmid (Addgene plasmid #136305), which presents the Cas9 and gRNAs expression cassettes [79]. Each primer had 5’ overhangs to the SapI enzyme cleavage site, which allowed the primers to be cloned into the plasmid. To do this assembly, each primer was first phosphorylated at the 5’-end with the polynucleotide kinase (PNK) enzyme for 1 h at 37 °C and then each pair of phosphorylated primers was pooled in a 1:10 dilution to perform the annealing procedure in a thermocycler at 96 °C for 6 min, lowering to 23 °C at a rate of 0.1 °C/second. With the primers hybridized, a Golden Gate procedure was performed, using a reaction containing 1 µL of SapI (NEB, USA), 1 µL of T4 DNA ligase (Thermo Scientific, USA), 1 µL 10X ligase buffer (NEB, USA), 0.5 µL of pAEF5 vector, and 4.5 µL of nuclease-free water. The reaction was incubated in a thermocycler for 10 cycles at 42 °C for 2 min and 16 °C for 5 min, then heated at 60 °C for 10 min and 80 °C for 10 min. Finally, 5 µL of the product was used to transform competent *E. coli* DH5α (Thermo Fisher, USA). A single colony was selected for plasmid purification.

Given the ability of Cas9 to generate double-stranded cuts, it is possible to repair the cleavage site with the endogenous yeast homologous recombination system [40]. For the replacement DNA fragment, sequences with the desired point mutation(s) and 50 bp homology upstream and downstream to the region of interest were synthesized. The linear fragment synthesized was amplified by PCR and the BY4741 strain was co-transformed with the plasmid containing the gRNA for the corresponding cut. The correct changes in the sequence of the *RRT5* and *PDR12* genes were confirmed by PCR amplification and sequencing of the PCR product (Macrogen, Republic of Korea).

## Electronic supplementary material

Below is the link to the electronic supplementary material.

Supplementary Material 1

Supplementary Material 2

## Data Availability

The datasets supporting the conclusions of this article are included within the article (and its additional files).

## Declarations

**Ethics approval and consent to participate**.

**Consent for publication**.

## Abbreviations

AA: Amino acid
ABC: ATP-binding cassette
ANOVA: Analysis of variance
ARS: Autonomously replicating sequence
AUC: Area under the curve
CNV: Copy number variant
FPM: Fermentation production medium
GM: Genetically modified
GWAS: Genome-wide association study
LOF: Loss-of-function
MAF: Minor allele frequency
NBT: New breeding technique
ORF: Open reading frame
QTL: Quantitative trait locus
SGD: *Saccharomyces* genome database
SM: Synthetic must
SNP: Single nucleotide polymorphism
WT: Wild type
YAN: Yeast assimilable nitrogen
