# Supplementary material for: Single nucleotide polymorphisms associated with wine fermentation and adaptation to nitrogen limitation in wild and domesticated yeast strains
Source: Biol Res. 2023 Jul 29;56:43. doi: 10.1186/s40659-023-00453-2 (PMC10385942; doi:10.1186/s40659-023-00453-2)
Supplement: Supplementary file 1 — Supplementary Material 1 [file 40659_2023_453_MOESM1_ESM.pdf]

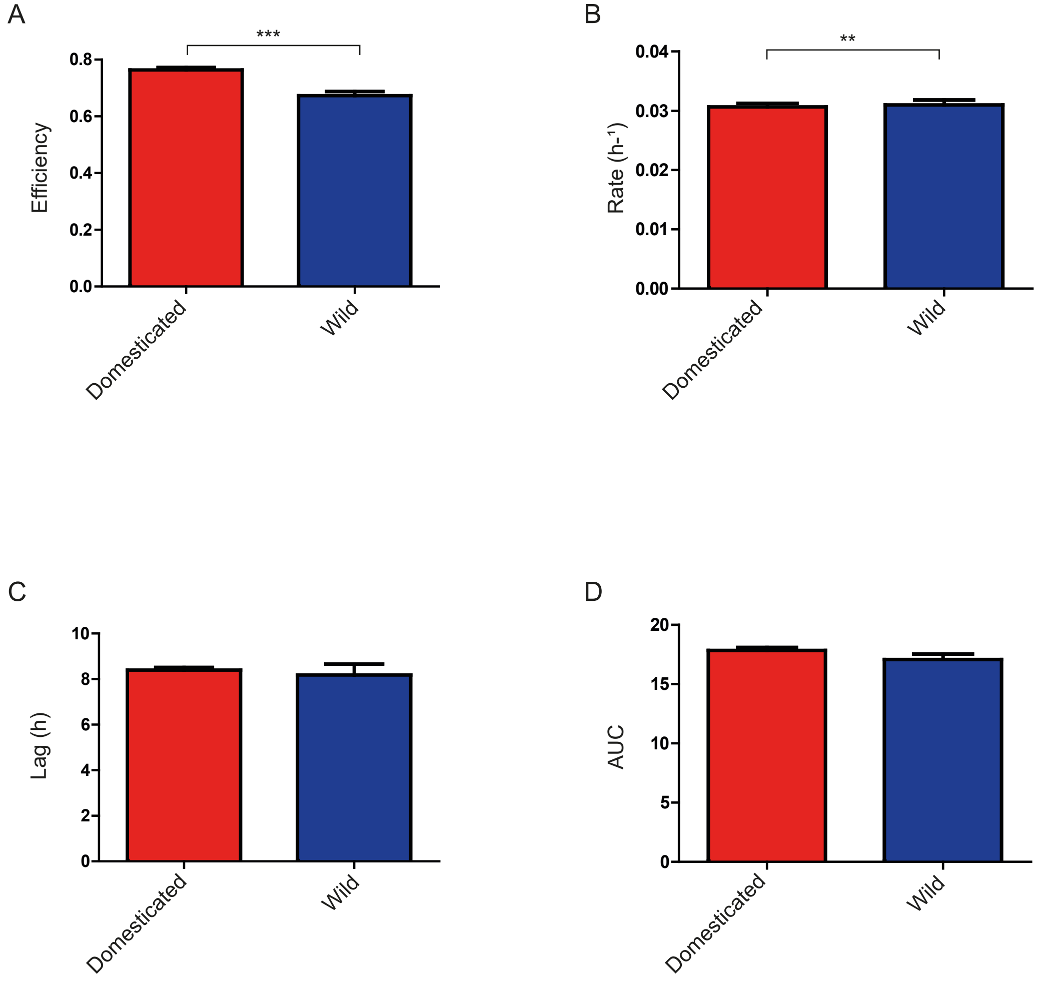

Figure S1. Comparison between domesticated and wild strains in SM300. The kinetic parameters compared were (A) efficiency, (B) rate, (C) lag and (D) AUC. Statistical analyses correspond to two-tailed Mann Whitney tests. ***: p<0.001, **: p<0.01, *: p<0.05.

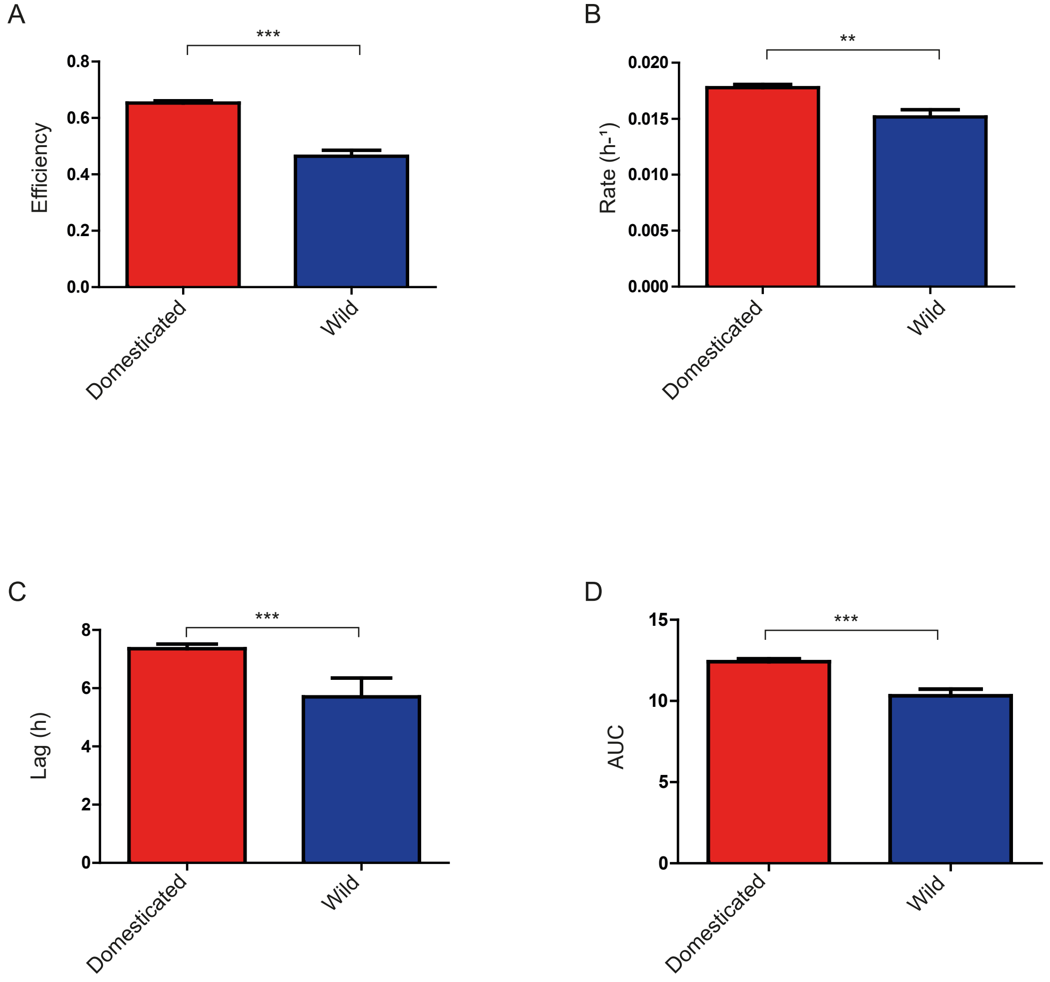

Figure S2. Comparison between domesticated and wild strains in SM60. The kinetic parameters compared were (A) efficiency, (B) rate, (C) lag and (D) AUC. Statistical analyses correspond to two-tailed Mann Whitney tests. ***: $p<0.001$, **: $p<0.01$, *: $p<0.05$.

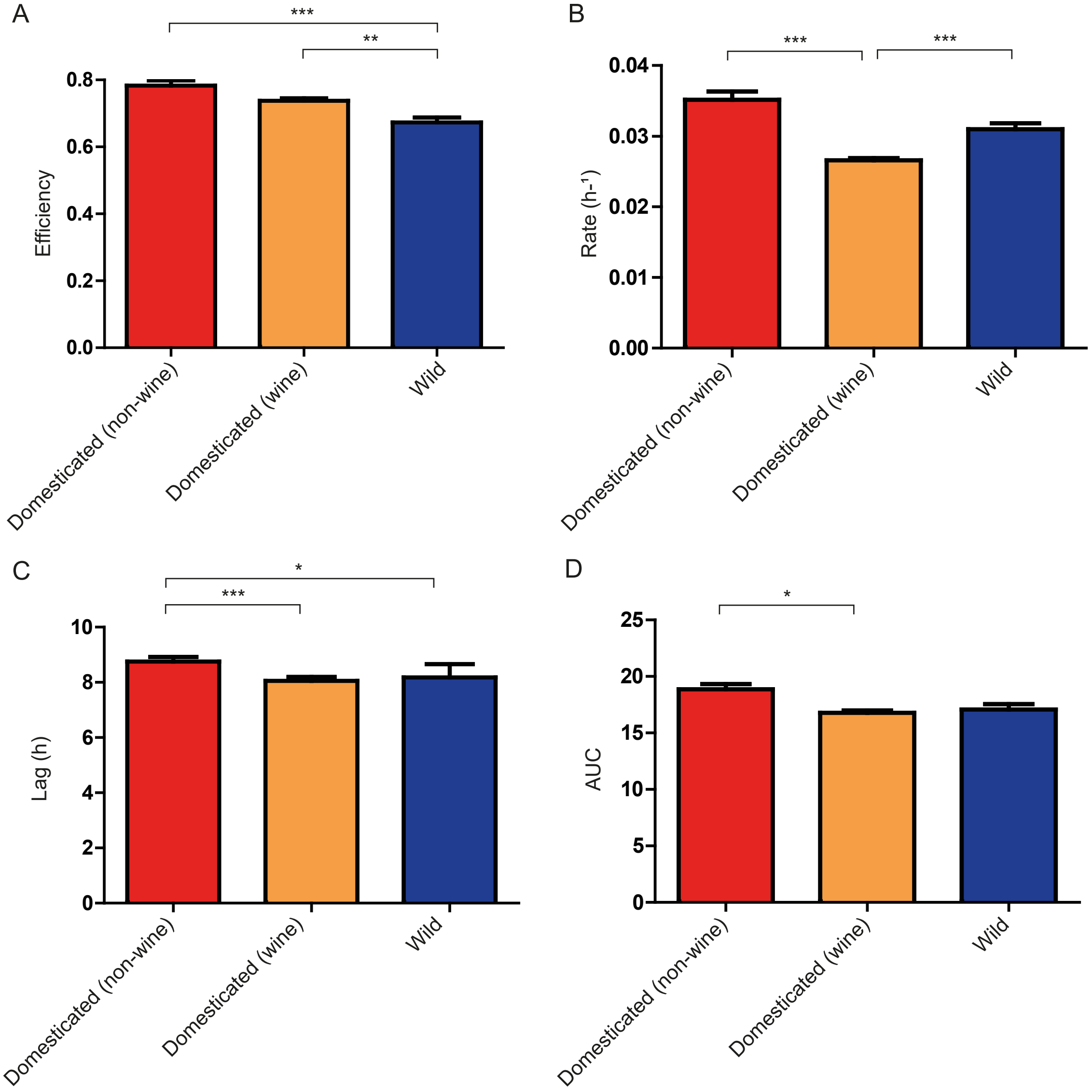

Figure S3. Comparison between domesticated (non-wine), domesticated (wine) and wild strains in SM300. The kinetic parameters compared were (A) efficiency, (B) rate, (C) lag and (D) AUC. Statistical analyses correspond to Kruskal-Wallis tests using Dunn's multiple comparisons tests. ***: $p<0.001$, **: $p<0.01$, *: $p<0.05$.

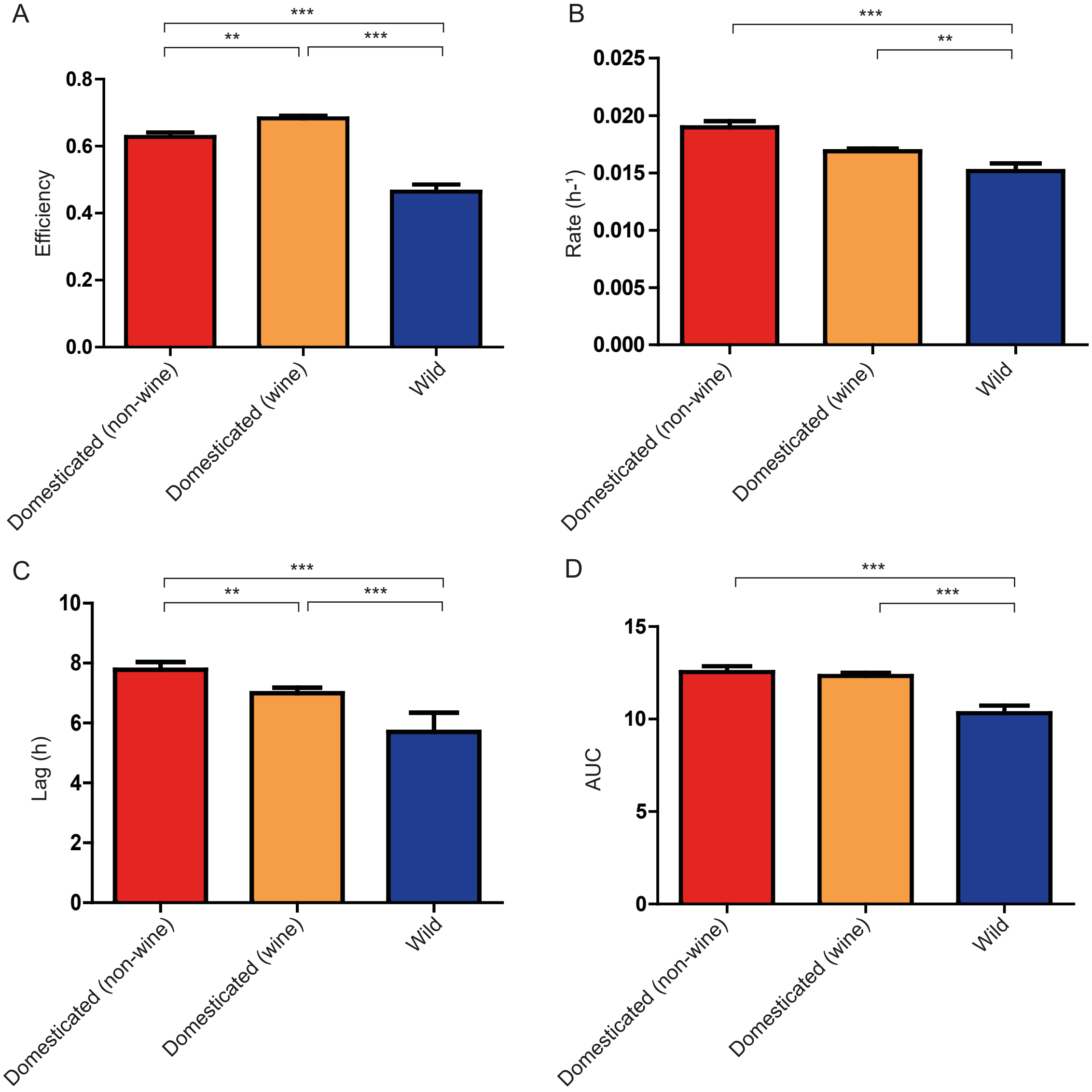

Figure S4. Comparison between domesticated (non-wine), domesticated (wine) and wild strains in SM60. The kinetic parameters compared were (A) efficiency, (B) rate, (C) lag and (D) AUC. Statistical analyses correspond to Kruskal-Wallis tests using Dunn's multiple comparisons tests. ***: $p<0.001$, **: $p<0.01$, *: $p<0.05$.

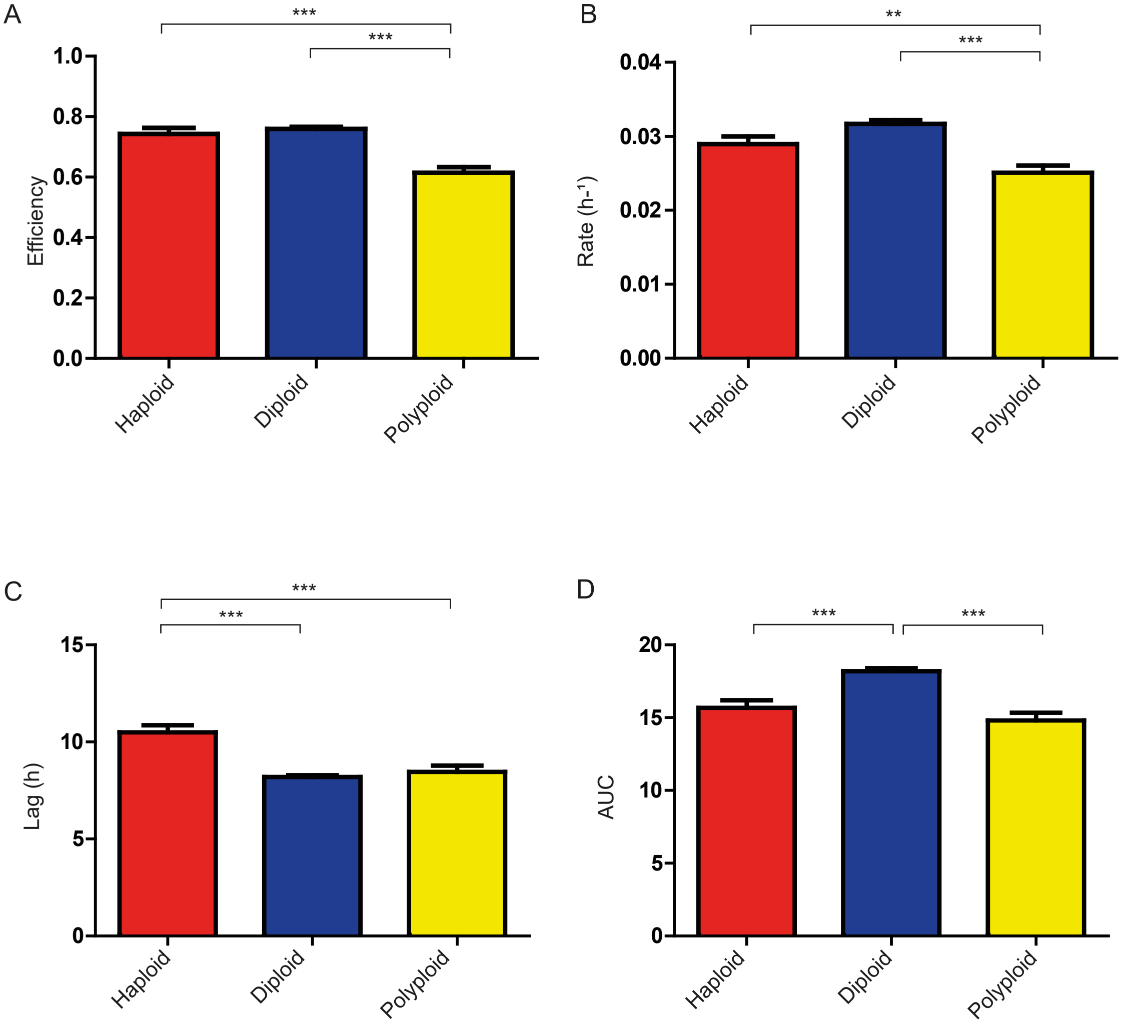

Figure S5. Comparison between haploid, diploidsand polyploid strains in SM300. The kinetic parameters compared were (A) efficiency, (B) rate, (C) lag and (D) AUC. Statistical analyses correspond to Kruskal-Wallis tests using Dunn's multiple comparisons tests.
***: $p<0.001$, **: $p<0.01$, *: $p<0.05$.

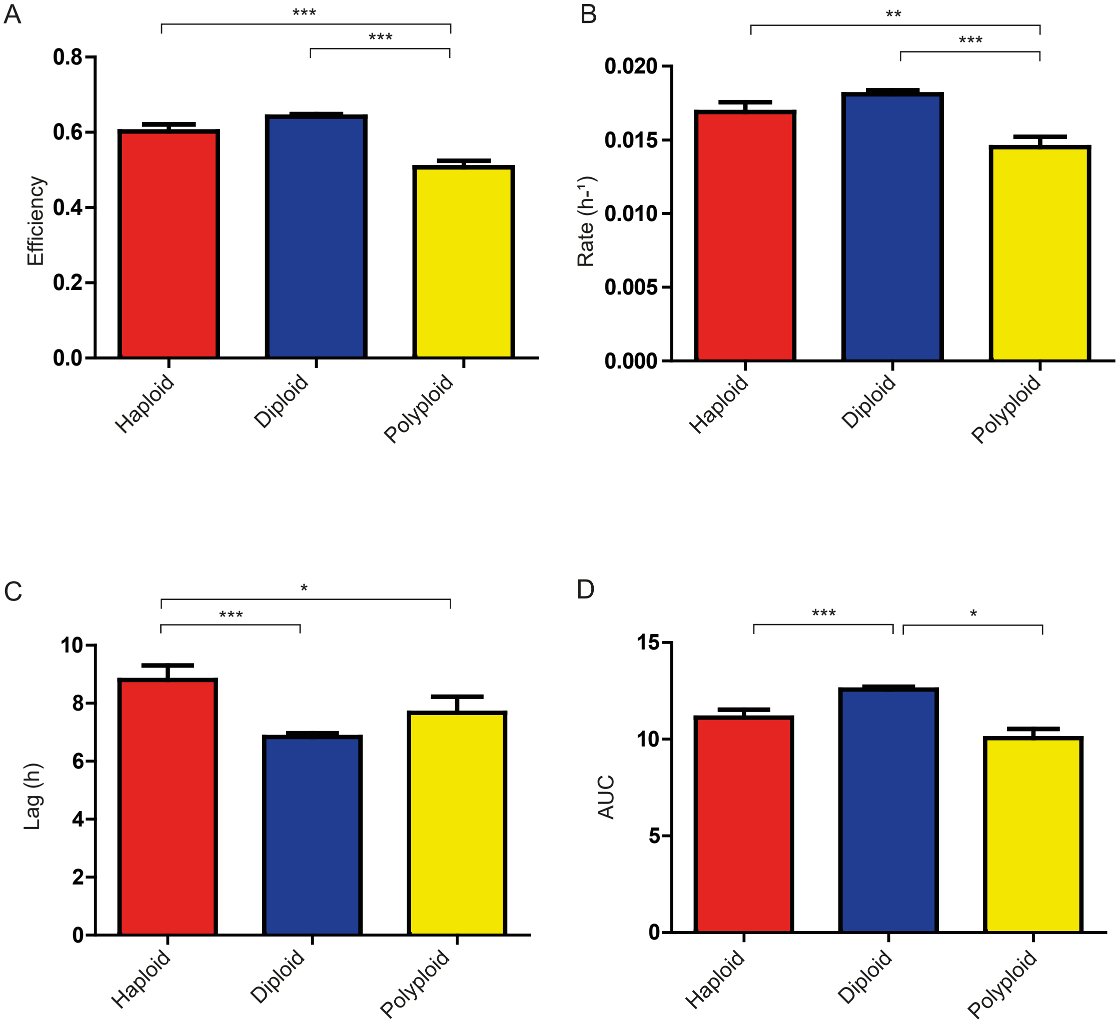

Figure S6. Comparison between haploid, diploid and polyploid strains in SM60. The kinetic parameters compared were (A) efficiency, (B) rate, (C) lag and (D) AUC. Statistical analyses correspond to Kruskal-Wallis tests using Dunn's multiple comparisons tests.
***: p<0.001, **: p<0.01, *: p<0.05.

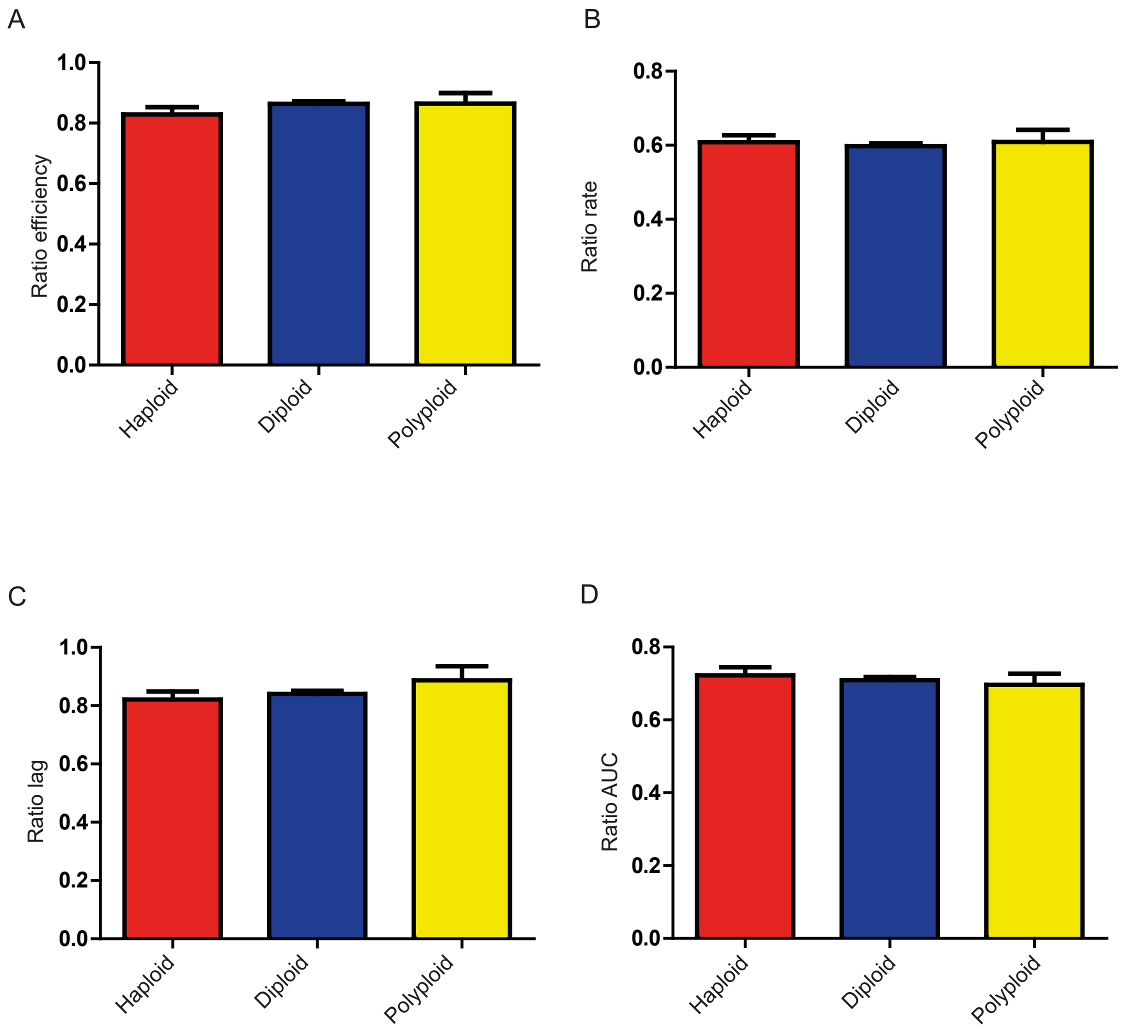

Figure S7. Comparison between haploid, diploid and polyploid strains for SM60/SM300 ratio. The kinetic parameters compared were (A) efficiency, (B) rate, (C) lag and (D) AUC. Statistical analyses correspond to Kruskal-Wallis tests using Dunn's multiple comparisons tests. ***: $p<0.001$, **: $p<0.01$, *: $p<0.05$.

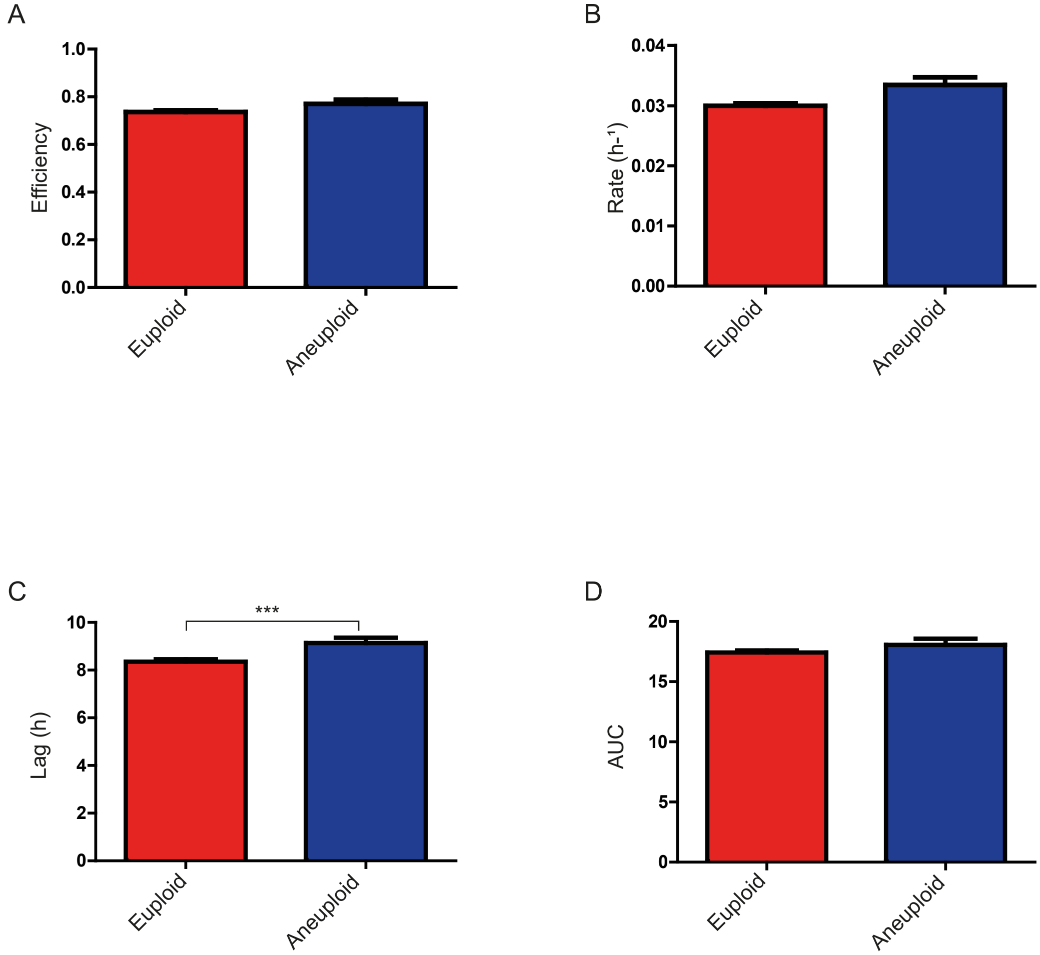

Figure S8. Comparison between euploid and aneuploid strains in SM300. The kinetic parameters compared were (A) efficiency, (B) rate, (C) lag and (D) AUC. Statistical analyses correspond to two-tailed Mann Whitney tests. ***: $p<0.001$, **: $p<0.01$, *: $p<0.05$.

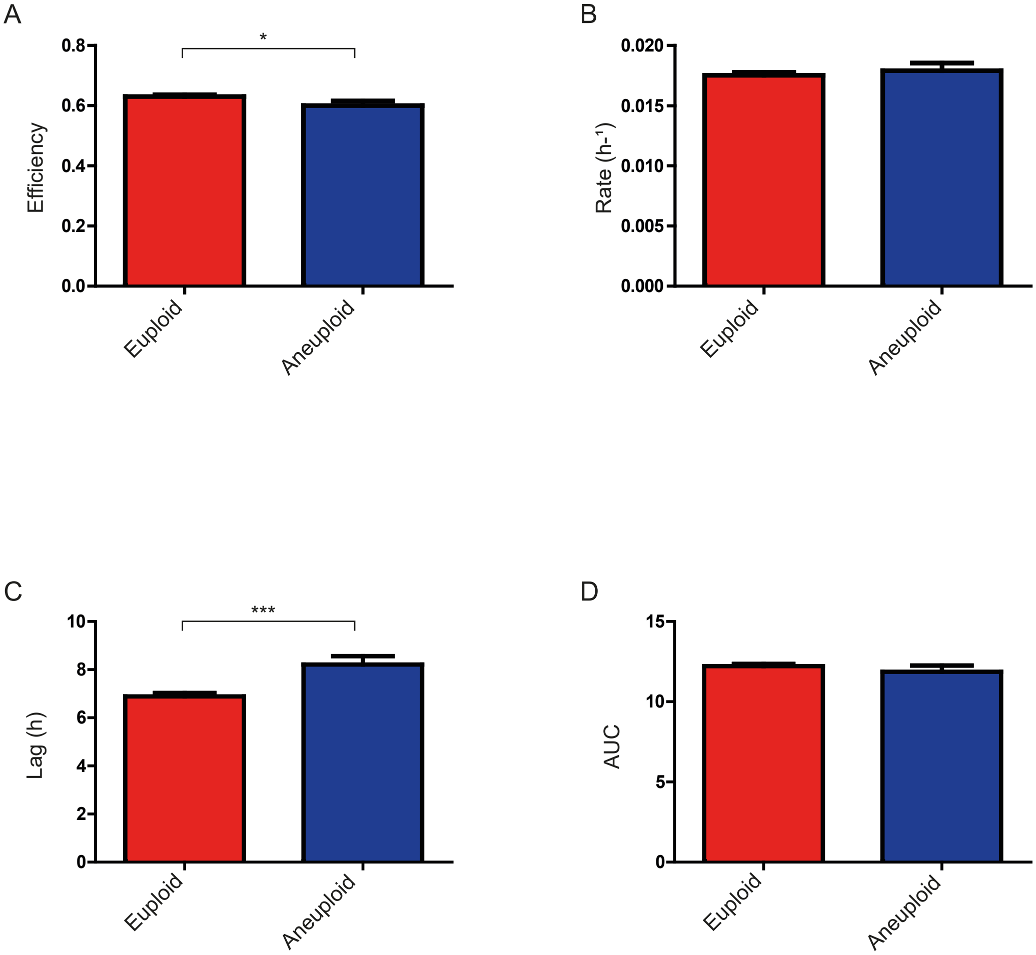

Figure S9. Comparison between euploid and aneuploid strains in SM60. The kinetic parameters compared were (A) efficiency, (B) rate, (C) lag and (D) AUC. Statistical analyses correspond to two-tailed Mann Whitney tests. ***: $p<0.001$, **: $p<0.01$, *: $p<0.05$.

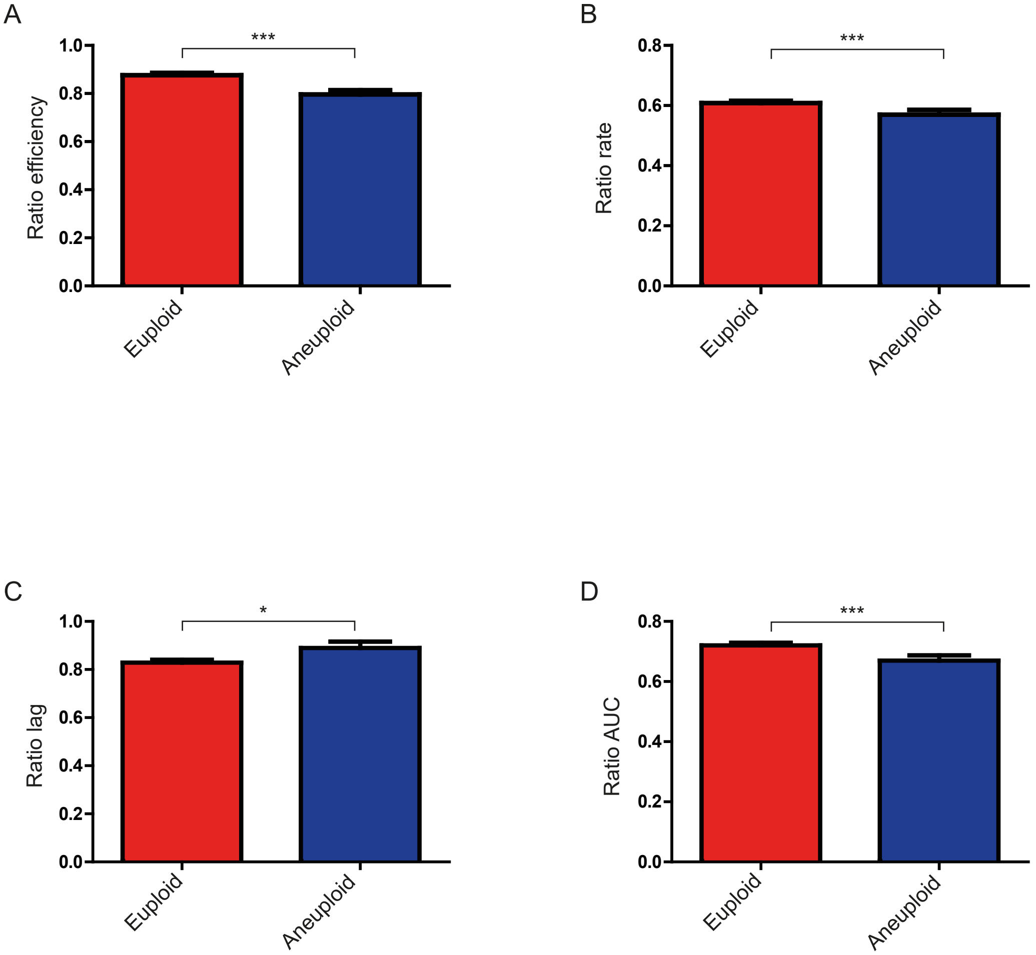

Figure S10. Comparison between euploid and aneuploid strains for the SM60/SM300 ratio. The kinetic parameters compared were (A) efficiency, (B) rate, (C) lag and (D) AUC. Statistical analyses correspond to two-tailed Mann Whitney tests. ***: $p<0.001$, **: $p<0.01$, *: $p<0.05$.

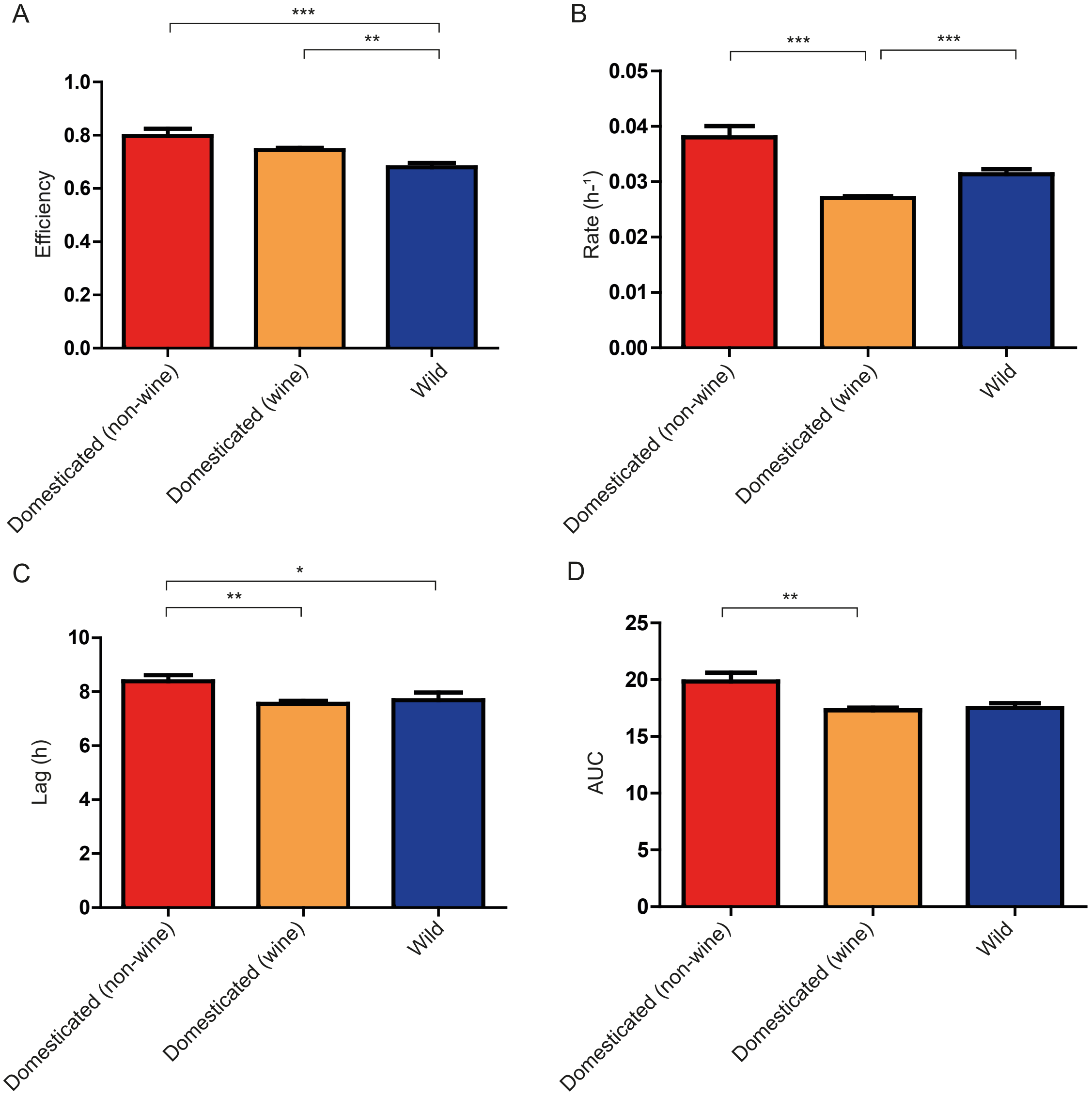

Figure S11. Comparison between domesticated (non-wine), domesticated (wine) and wild diploid-euploid strains in SM300. The kinetic parameters compared were (A) efficiency, (B) rate, (C) lag and (D) AUC. Statistical analyses correspond to Kruskal-Wallis tests using Dunn's multiple comparisons tests. ***: $p<0.001$, **: $p<0.01$, *: $p<0.05$.

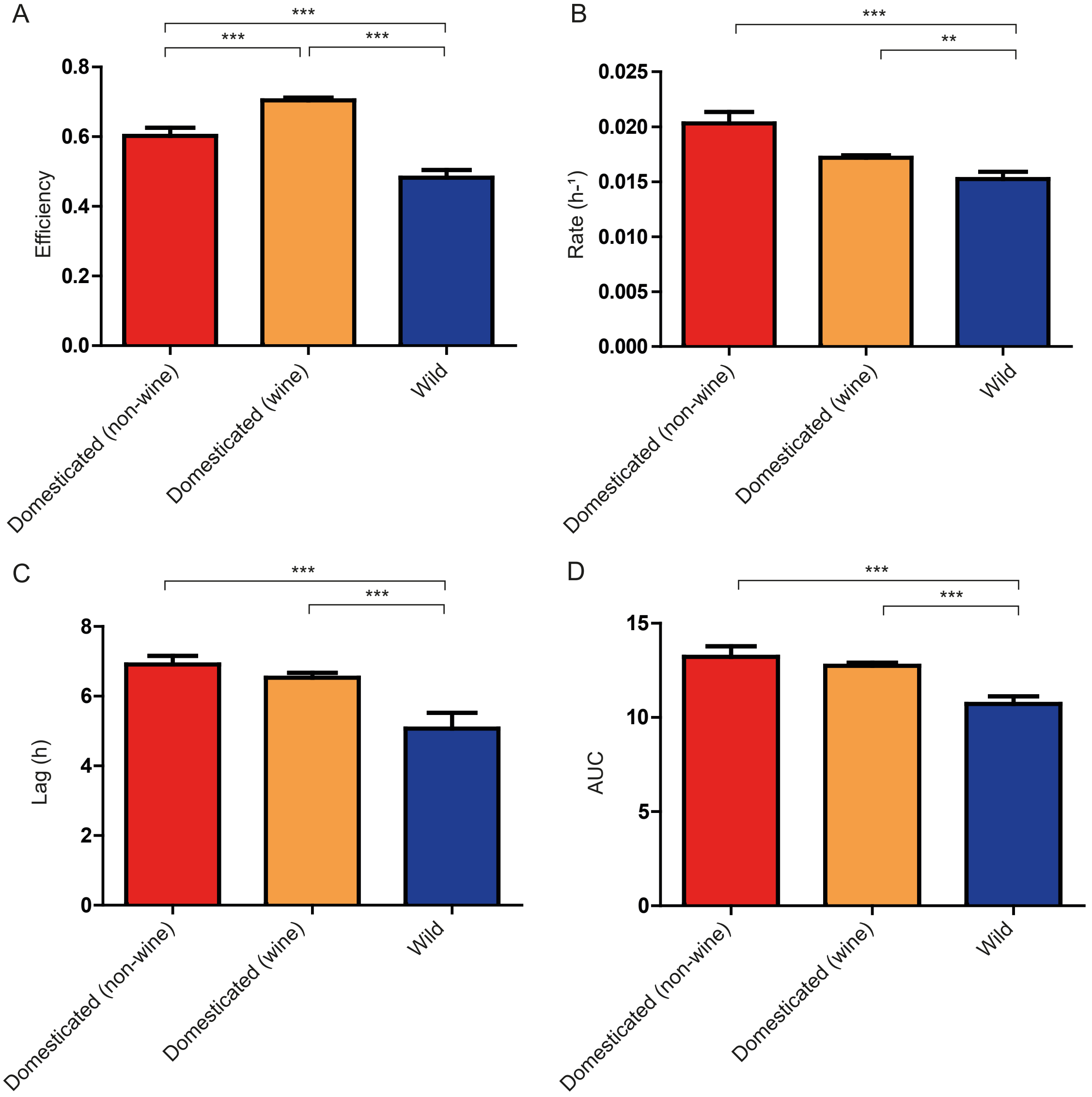

Figure S12. Comparison between domesticated (non-wine), domesticated (wine) and wild diploid-euploid strains in SM60. The kinetic parameters compared were (A) efficiency, (B) rate, (C) lag and (D) AUC. Statistical analyses correspond to Kruskal-Wallis tests using Dunn's multiple comparisons tests. ***: $p<0.001$, **: $p<0.01$, *: $p<0.05$.

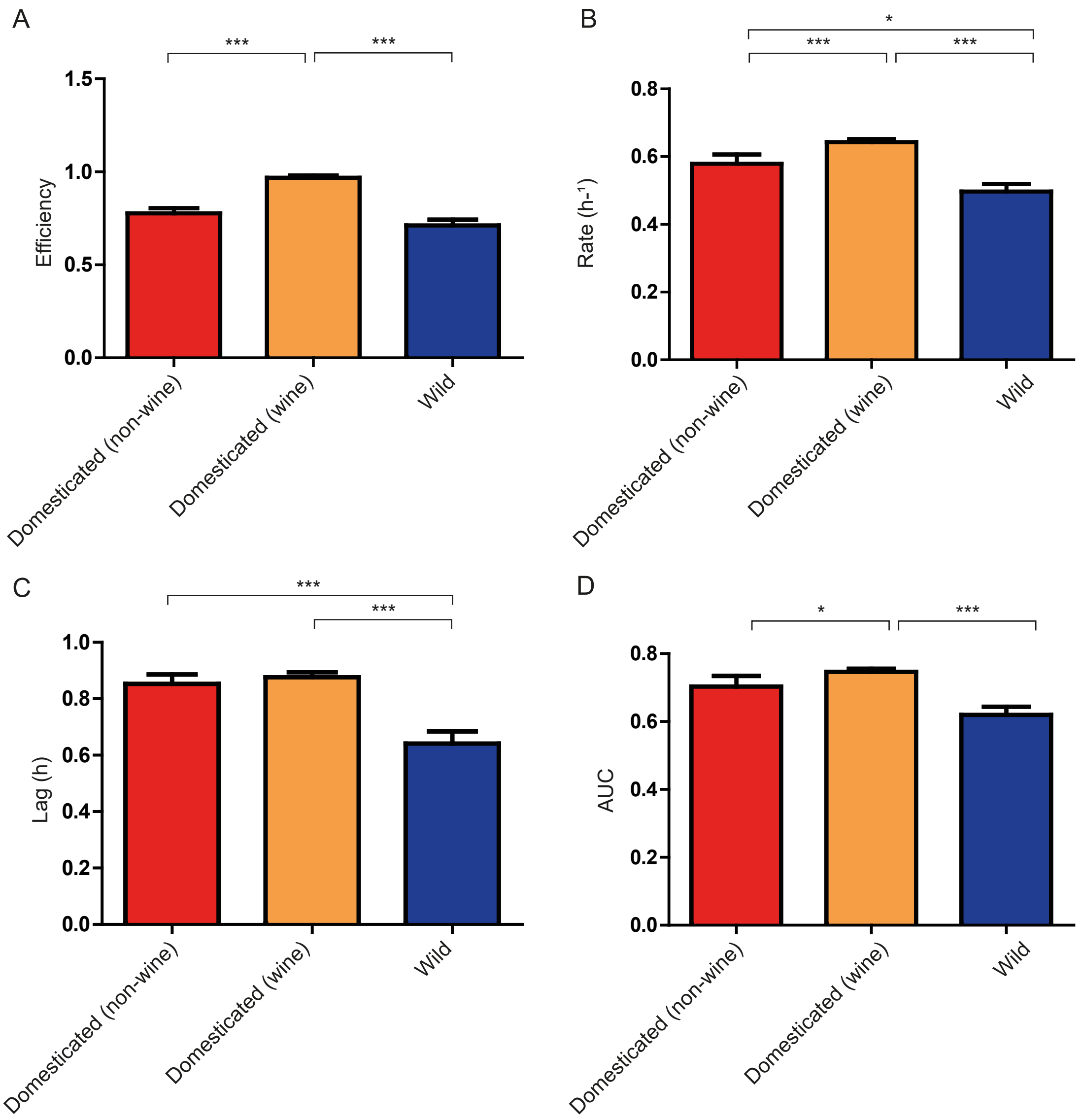

Figure S13. Comparison between domesticated (non-wine), domesticated (wine) and wild diploid-euploid strains for the SM60/SM300 ratio. The kinetic parameters compared were (A) efficiency, (B) rate, (C) lag and (D) AUC. Statistical analyses correspond to Kruskal-Wallis tests using Dunn's multiple comparisons tests. ***: $p<0.001$, **: $p<0.01$, *: $p<0.05$.

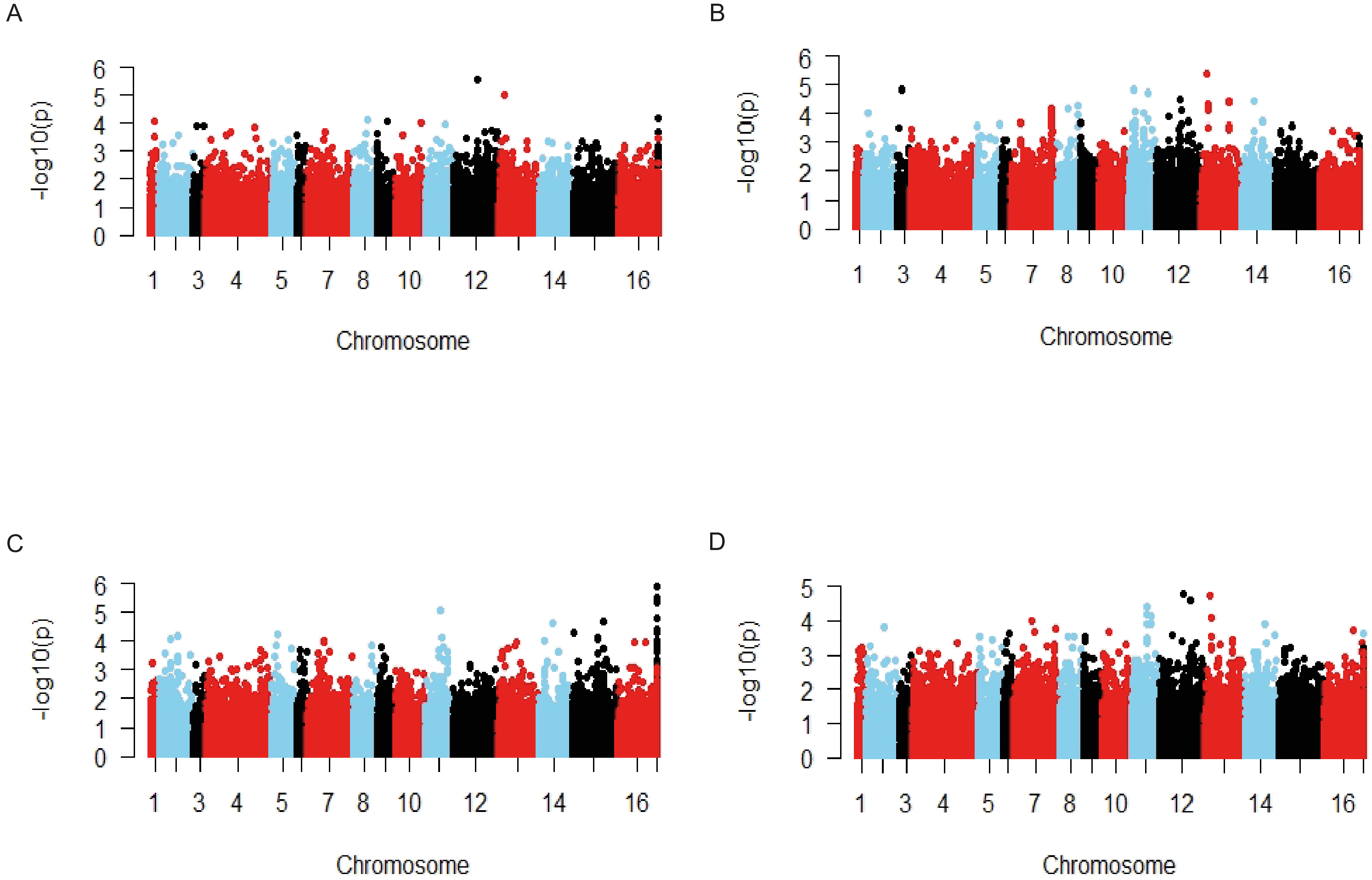

Figure S14. Manhattan plots of the GWAS performed with the phenotypes obtained in SM300. The phenotypic data used correspond to the kinetic parameters of (A) efficiency, (B) rate, (C) lag and (D) AUC.

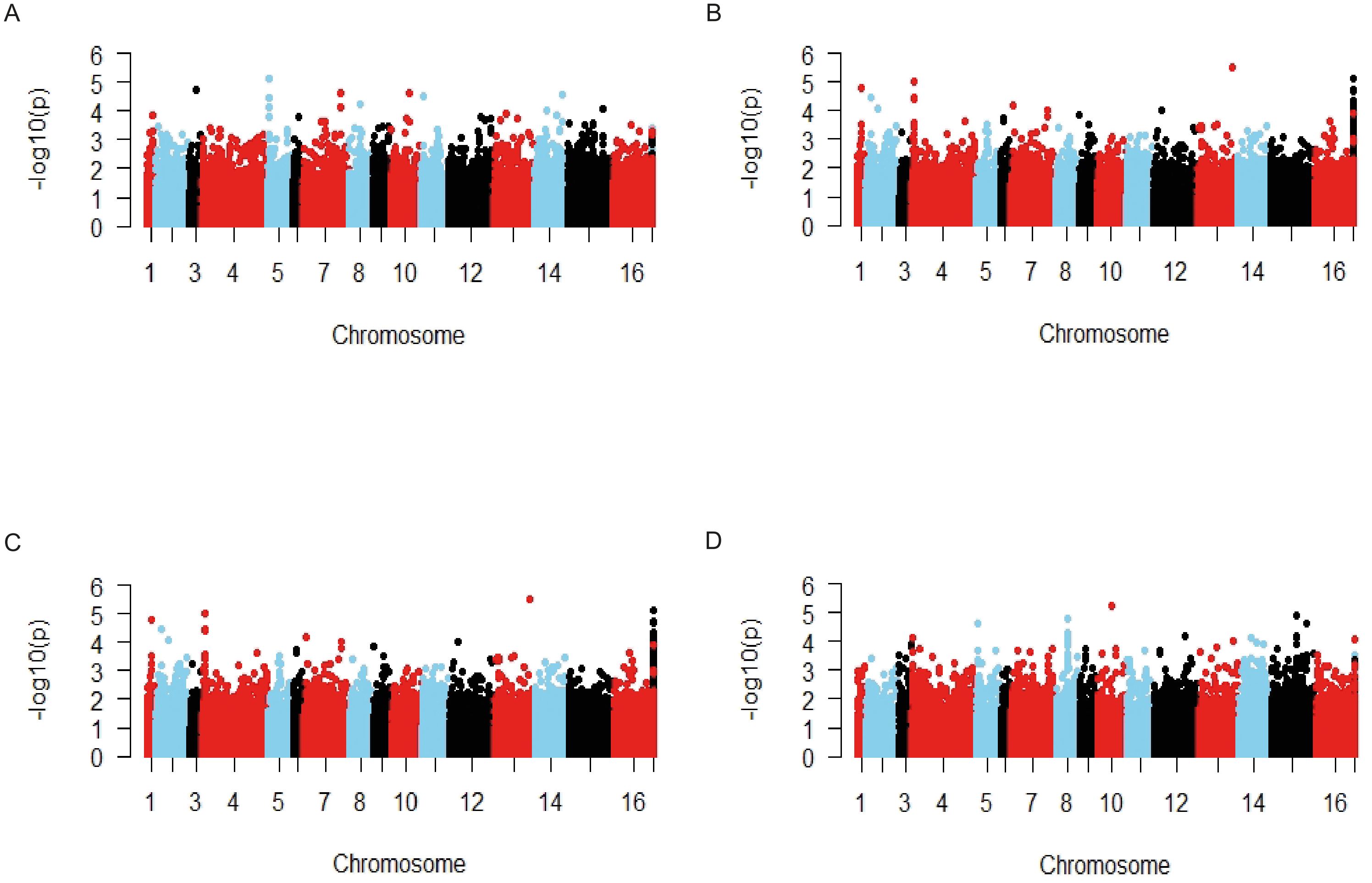

Figure S15. Manhattan plots of the GWAS performed with the phenotypes obtained in SM60. The phenotypic data used correspond to the kinetic parameters of (A) efficiency, (B) rate, (C) lag and (D) AUC.

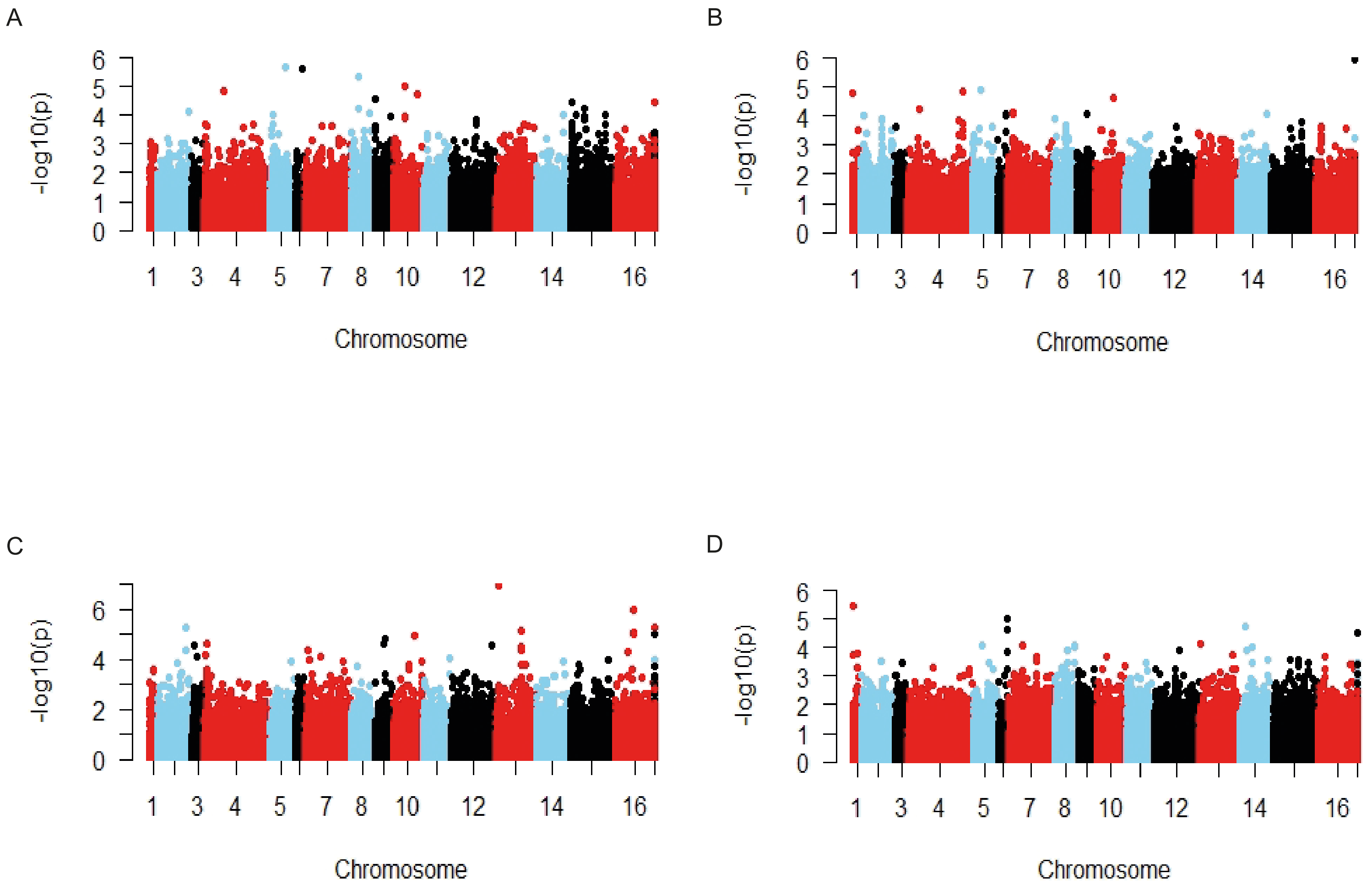

Figure S16. Manhattan plots of the GWAS performed with the phenotypes obtained for the SM60/SM300. The phenotypic data used correspond to the kinetic parameters of (A) efficiency, (B) rate, (C) lag and (D) AUC.

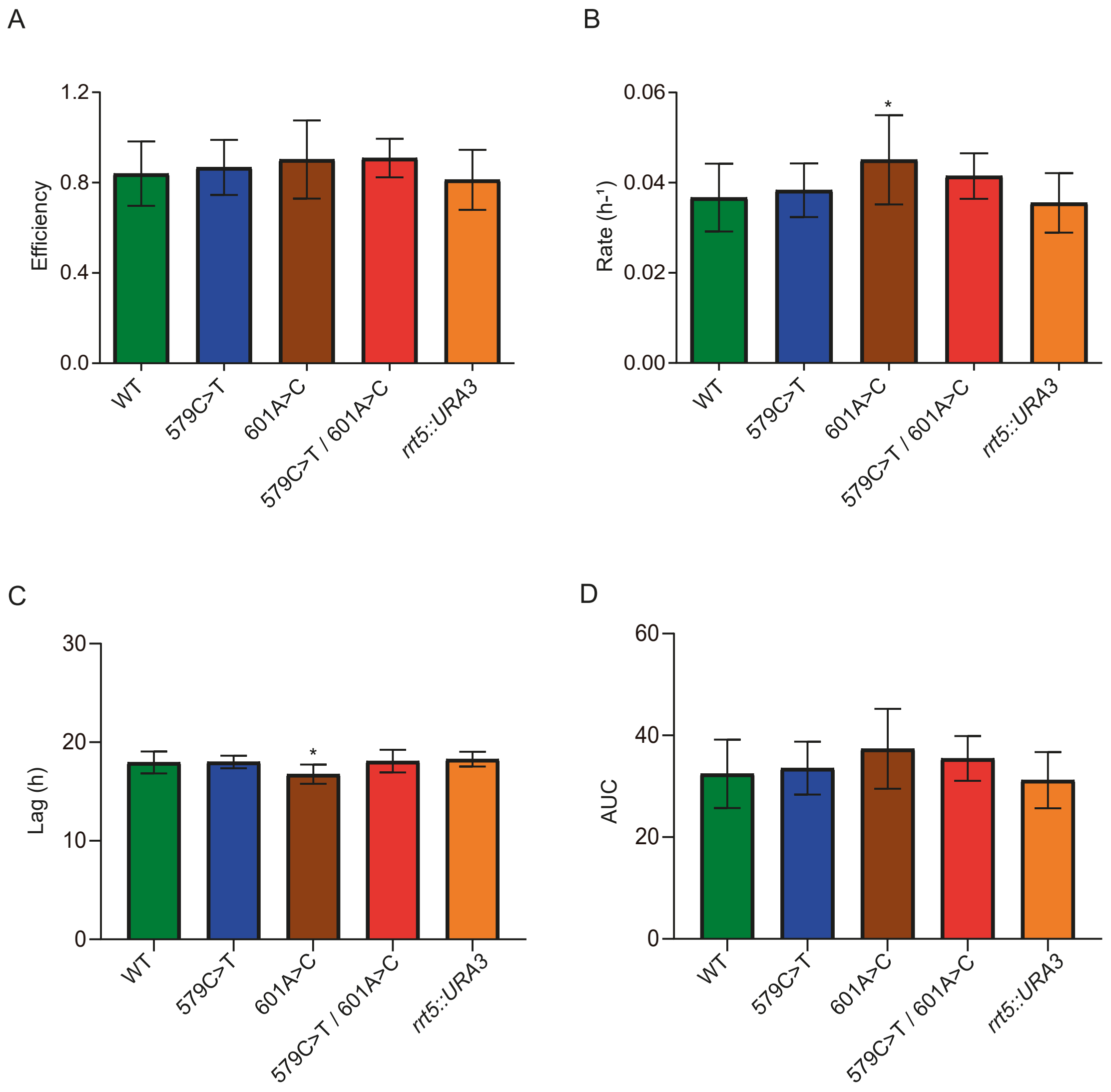

Figure S17. Comparison between *RRT5* mutants and their wild type (WT) strain in SM300. The kinetic parameters compared were (A) efficiency, (B) rate, (C) lag and (D) AUC. Statistical analyses correspond to ordinary one-way ANOVA using Holm-Šídák's multiple comparisons tests, comparing in each case the WT versus the different mutants. ***: p<0.001, **: p<0.01, *: p<0.05.

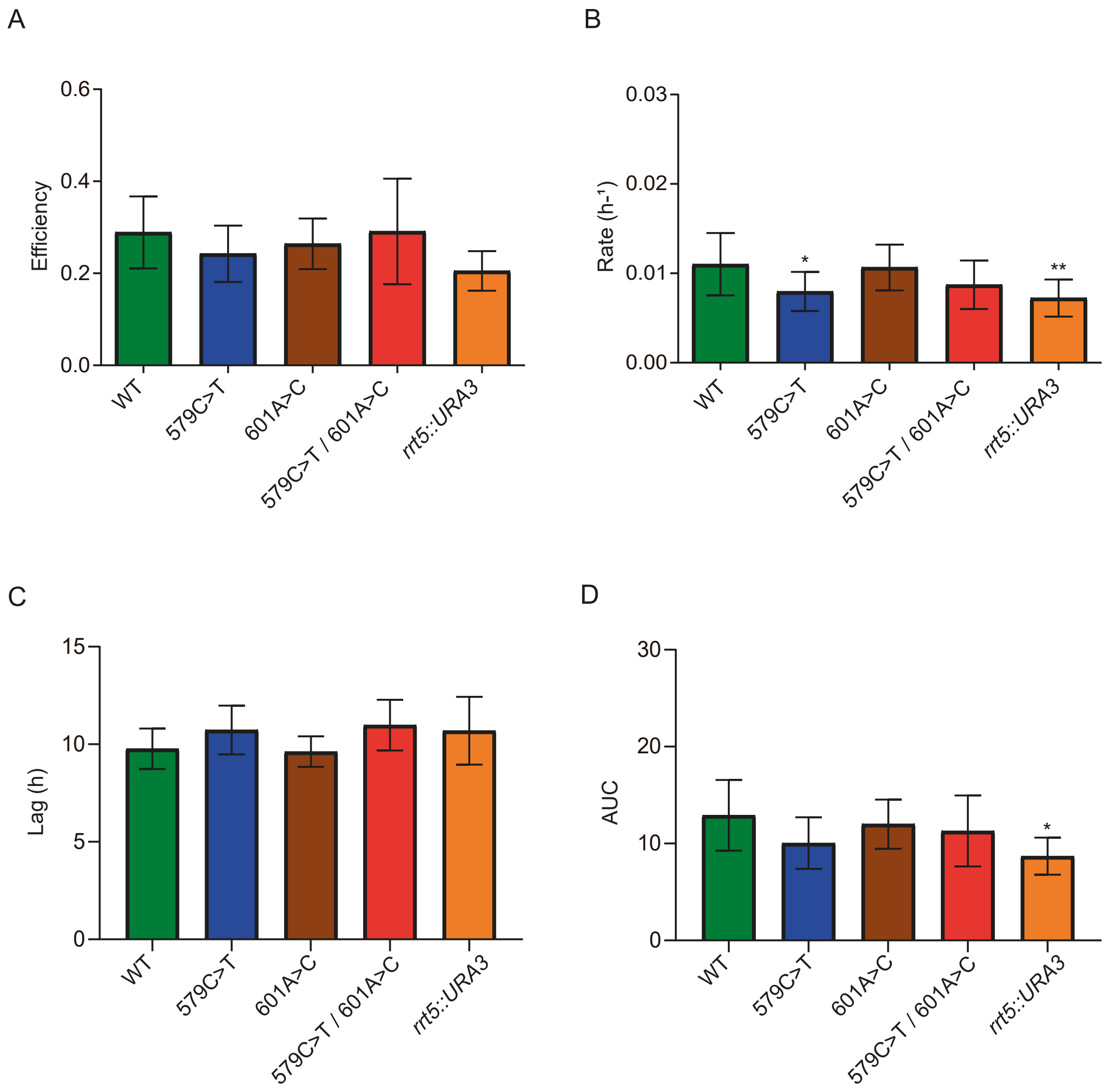

Figure S18. Comparison between *RRT5* mutants and their wild type (WT) strain in SM60. The kinetic parameters compared were (A) efficiency, (B) rate, (C) lag and (D) AUC. Statistical analyses correspond to ordinary one-way ANOVA using Holm-Šídák's multiple comparisons tests, comparing in each case the WT versus the different mutants. ***: $p<0.001$, **: $p<0.01$, *: $p<0.05$.

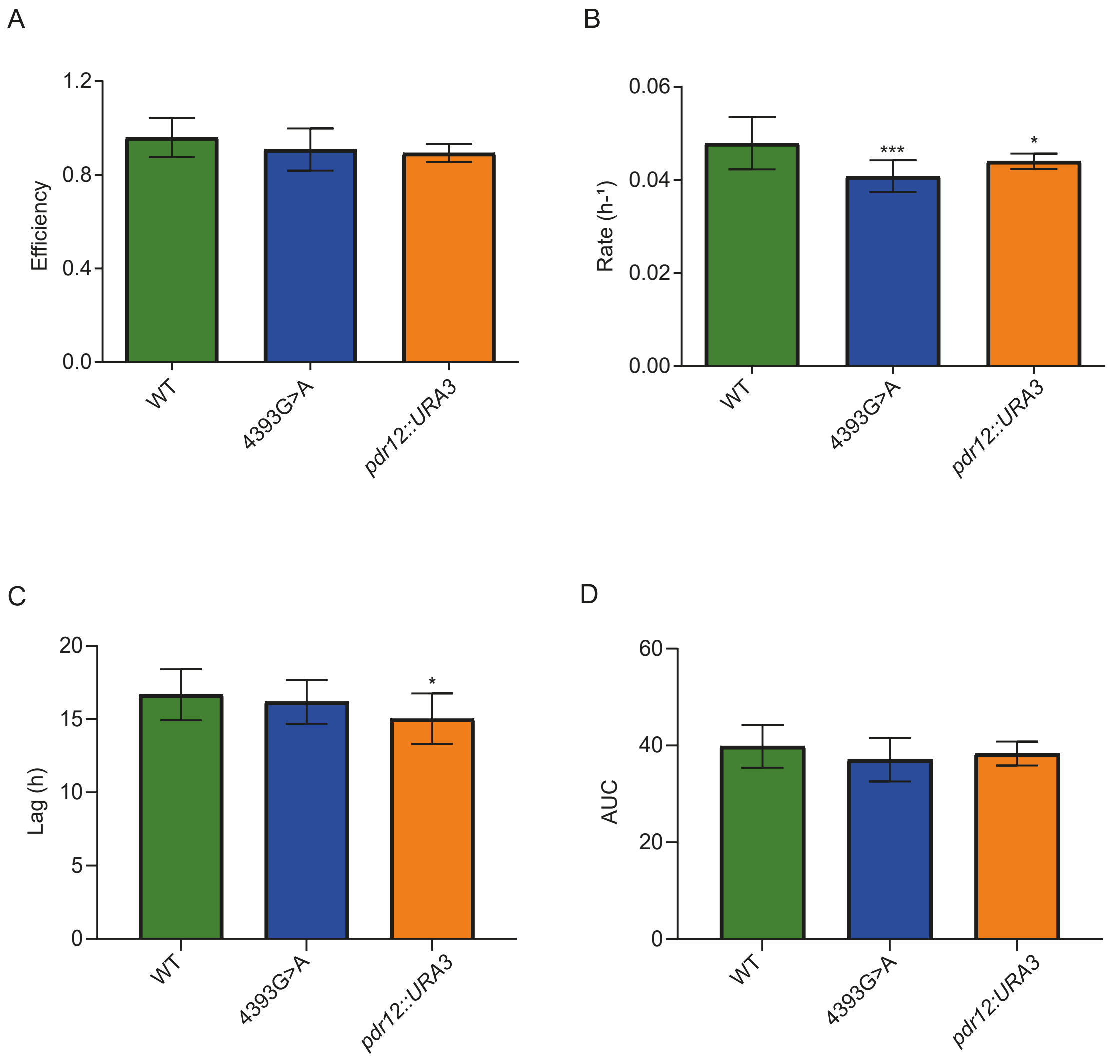

Figure S19. Comparison between *PDR12* mutants and their wild type (WT) strain in SM300. The kinetic parameters compared were (A) efficiency, (B) rate, (C) lag and (D) AUC. Statistical analyses correspond to ordinary one-way ANOVA using Holm-Šídák's multiple comparisons tests, comparing in each case the WT versus the different mutants. ***: $p<0.001$, **: $p<0.01$, *: $p<0.05$.

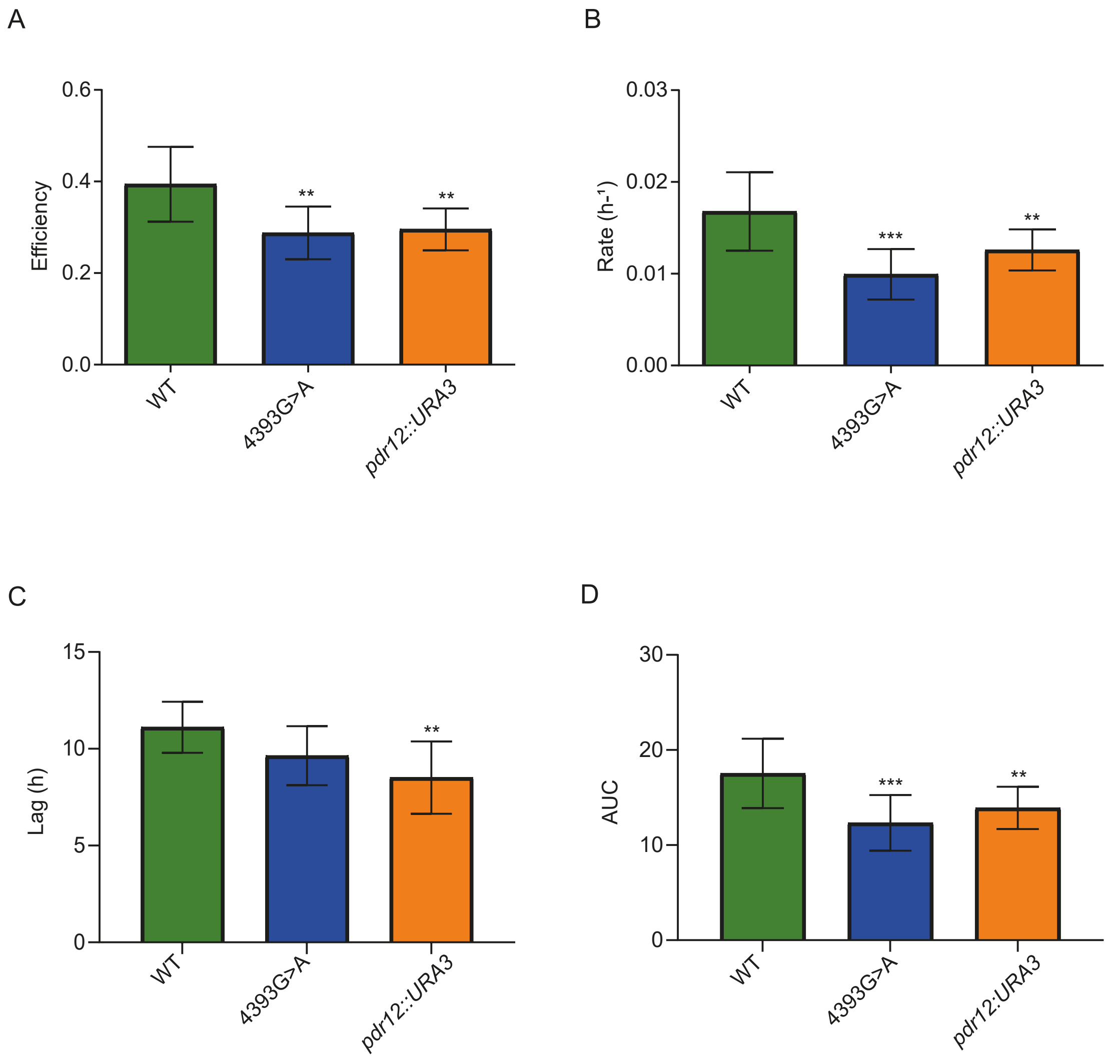

Figure S20. Comparison between *PDR12* mutants and their wild type (WT) strain in SM60. The kinetic parameters compared were (A) efficiency, (B) rate, (C) lag and (D) AUC. Statistical analyses correspond to ordinary one-way ANOVA using Holm-Šídák's multiple comparisons tests, comparing in each case the WT versus the different mutants. ***: $p<0.001$, **: $p<0.01$, *: $p<0.05$.
